# Sulfated Polysaccharides from Sea Cucumber Cooking Liquid Prevents Obesity by Modulating Gut Microbiome, Transcriptome, and Metabolite Profiles in Mice Fed a High-Fat Diet

**DOI:** 10.3390/foods13132017

**Published:** 2024-06-26

**Authors:** Xue Sang, Xin Guan, Yao Tong, Fuyi Wang, Boqian Zhou, Ying Li, Qiancheng Zhao

**Affiliations:** 1 College of Food Science and Engineering, Dalian Ocean University, Dalian 116023, China; sangxue116@hotmail.com (X.S.); guanxin0222@163.com (X.G.); tongyao03272024@163.com (Y.T.); wfy13009346433@163.com (F.W.); zhouboqian00@163.com (B.Z.); liying@dlou.edu.cn (Y.L.); 2 Dalian Key Laboratory of Marine Bioactive Substances Development and High-Value Utilization, Dalian 116023, China; 3 Liaoning Provincial Marine Healthy Food Engineering Research Centre, Dalian 116000, China; 4 Collaborative Innovation Center of Provincial and Ministerial Co-Construction for Marine Food Deep Processing, Dalian Polytechnic University, Dalian 116034, China

**Keywords:** sea cucumber cooking liquid, polysaccharides, managing obesity, gut microbiota, transcriptome, metabolomics

## Abstract

We aimed to explore the anti-obesity mechanism from the microbiome, metabolome, and transcriptome viewpoints, focusing on the sulfated polysaccharides found in the cooking liquid of *Apostichopus japonicus* (CLSP_AJ_) to explore the potential mediators of the anti-obesity effects in mice fed a high-fat diet (HFD). The mice treated with CLSP_AJ_ showed a decrease in obesity and blood lipid levels. Gut microbiome dysbiosis caused by the HFD was reversed after CLSP_AJ_ supplementation, along with increased levels of indole-3-ethanol, N-2-succinyl-L-glutamic acid 5-semialdehyde, and urocanic acid. These increases were positively related to the increased *Akkermansia*, *Lactobacillus*, *Roseburia*, and *Phascolarctobacterium*. Transcriptome analysis showed that B cell receptor signaling and cytochrome P450 xenobiotic metabolism were the main contributors to the improvement in obesity. Metabolome–transcriptome analysis revealed that CLSP_AJ_ reversal of obesity was mainly due to amino acid metabolism. These findings suggest that CLSP_AJ_ could be a valuable prebiotic preparation for preventing obesity-related diseases.

## 1. Introduction

Obesity caused by unhealthy eating habits and lifestyles has grown to be a global public health problem, with excess body mass index being the fifth leading risk factor for death globally. Obesity typically leads to health complications, including diabetes, hyperlipidemia, certain gastrointestinal disorders, and metabolic syndrome [1]. Obesity represents an energy imbalance due to a chronic excess of calorie intake over energy expenditure in the modern environment. The imbalance between the two occurs in the intestine. Analysis of altered intestinal flora in a young obese Chinese Han population suggested that the intestinal bacterial diversity in this population was markedly reduced and that changes in intestinal flora may also be involved in the weight loss effects of bariatric surgery [2]. In the food preference tests, *Parabacteroides* was identified as a potential participant in hedonic dietary intake through the gut–brain axis, demonstrating that gut microbes may be a therapeutic target to address obesity-related issues [3]. The findings suggest that the gut micro-ecosystem is a bridge between the external and internal environment of the organism, capable of breaking down food, sensing nutrient status, assisting energy absorption, regulating host metabolism and immunity, and interacting with the host to maintain host energy metabolic homeostasis, and is a newly identified factor influencing obesity [4].

Sea cucumber (*Apostichopus japonicus*) is a highly prized seafood that has been consumed in Asian countries for over a thousand years, and its nutritional effects have been of great interest [5]. The commercial processing of sea cucumber is accompanied by a large amount of cooking waste, resulting in environmental pollution and a waste of biological resources. It is therefore desirable to utilize this waste because it contains large amounts of polysaccharides which have high economic value upon extraction from sea cucumber processing liquid [6].

Due to their sulfated group and special structures, marine-sulfated polysaccharides have been reported to possess a range of biological activities [7]. Moreover, the source of the SPs, the type of monosaccharides and glycosidic bonds, the molecular weights, and the important positions and degrees of sulfation can lead to significant differences in biological activities [8]. Various studies have confirmed that different types of polysaccharides can act as prebiotics to restore obesity-induced metabolic disorders by regulating the gut microbiome, which in turn affects nutrient acquisition and energy regulation [9]. Considering the diverse effects of different polysaccharide types on the gut microbiota, metabolites and related genes, the mechanism of how CLSP_AJ_ regulates obesity is unclear.

This study aimed at exploring the mechanism of obesity prevention by CLSP_AJ._ Firstly, we used biochemical analysis and histological examination in HFD-fed mice to demonstrate that CLSP_AJ_ was effective in ameliorating HDF-induced obesity. Secondly, we assessed the alterations in the gut microbiome and resulting metabolite changes after CLSP_AJ_ administration. Potential relationships between changes in the gut microbiome and its metabolites were discussed to enable the identification of candidate microorganisms and metabolites. Finally, transcriptome analysis in the liver of the mice was carried out. This study elucidated the anti-obesity mechanism of CLSP_AJ_ and provided a theoretical basis and reference for the development and application of CLSP_AJ_ in functional foods.

## 2. Materials and Methods

### 2.1. Preparation of CLSP_AJ_

The live sea cucumbers were captured in the Dalian Bohai Sea, and the sea cucumber cooking liquid (CL_AJ_) was obtained by cooking sea cucumber body walls in water at 85–90 °C for 15 min. The CLSP_AJ_ was created according to the published method [10]. Briefly, the CL_AJ_ was placed in triple (*v*/*v*) anhydrous ethanol and left for 24 h at 4 °C, followed by centrifugation at 6000× *g* for 15 min, after which the pellet was collected and lyophilized. We accurately weighed 100 g of lyophilized crude polysaccharides, added 2 L of 0.1 mol/L sodium acetate buffer solution (pH 6.0), followed by adding 2.64 g cysteine hydrochloride and 4.38 g EDTA. Then, the pH was adjusted to 6.0 using acetic acid and 10 g papain was added; the enzymatic digestate was stirred at 60 °C for 24 h, centrifuged at 6000× *g* for 20 min, and the supernatant was collected. Afterward, the samples were mixed with 3 times (*v*/*v*) ethanol for polysaccharide precipitation. After centrifugation at 4 °C 6000× *g* for 15 min, the pellet was dissolved and dialyzed against distilled water (3 kDa molecular weight cut-off). The CLSP_AJ_ was obtained by lyophilizing, and the rate of extractable sulfated polysaccharides in the sea cucumber cooking liquid was 1.74 g/L. 

### 2.2. Compositional Analysis of CLSP_AJ_

The contents of the sulfated group, uronic acid, and protein were measured according to Dodgson and Price [11], Filisetti-Cozzi and Carpita [12], and Bradford [13], respectively. Gel permeation chromatography (GPC) on the TSK G4000PWXL column (7.5 × 300 mm^2^) and refractive index detector (RID-10A, SHIMADZU, Tokyo, Japan) was used to determine the molecular weight (MW) distribution of CLSP_AJ_ using a series of dextran standards.

The monosaccharide composition of CLSP_AJ_ was analyzed by HPLC after trifluoroacetic acid hydrolyzation and 1-phenyl-3-methyl-5-pyrazolone derivatization using a 20 μL sample loop and an Agilent C18 (5 μm, 4.6 × 250 mm^2^) column.

According to the reported method [14], the functional groups present in compound CLSP_AJ_ were characterized using Fourier Transform Infrared Spectroscopy (FT-IR) (Xi’an HEB Biotechnology Co., Xi’an, China).

Dried CLSP_AJ_ was weighed (5–10 mg) into a solid crucible that was sealed tightly using a press. Thermodynamic properties were determined using a differential scanning calorimeter (Q20, TA Instruments, NewCastle, DE, USA) at scanning temperatures of 40–400 °C, with a temperature rise rate of 10 °C/min.

### 2.3. Animals and Experimental Design

A total of 36 six-week-old C57BL/6J male mice were acquired from Liaoning Changsheng Biotechnology Co., Ltd., Benxi, China (Permission Number: SCXK2020-0001). Mice were randomly assigned to individually ventilated cages (nine mice/group and three mice/cage) with light/dark cycling for 12 h under standard animal laboratory conditions (22 ± 2 °C and relative humidity 50 ± 10%). The mice were randomized into four groups after 1 week of acclimatization on a normal diet; one group was fed a normal maintenance diet (Control group, D12450B, containing 10% kcal from fat), and one group was fed an HFD (HFD group, D12451B, containing 45% kcal from fat) as the obesity model group. The control and HFD groups were given saline by gavage, and the HFD_SP_L and HFD_SP_H groups were fed an HFD diet and given the dose of 250 mg·kg^−1^·d^−1^ and 500 mg·kg^−1^·d^−1^ of CLSP_AJ_, respectively [15]. During the 8-week supplementation, food consumption and body weight were measured daily. After fasting for 12 h, all mice were anesthetized with ether at the end of an 8-week period, and then orbital blood was collected. The mice were then executed and underwent steps such as organ dissection and weighing. 

The Animal Experiments Committee on the Ethics of Obio Technology (Shanghai) Corp., Ltd., Shanghai, China, approved all animal protocols according to National Research Council ethical guidelines (Approval number: 112) in this study.

### 2.4. Biochemical Analysis of Serum Physiological Indices

The blood was centrifuged (4000 rpm, 20 min) to obtain serum, and total cholesterol (T-CHO), triacylglycerols (TG), high-density lipoprotein cholesterol (HDL-C), low-density lipoprotein cholesterol (LDL-C), malondialdehyde (MDA), alanine aminotransferase (ALT), and aspartate aminotransferase (AST) were measured using commercial kits (Jiancheng Bioengineering Institute, Nanjing, China). 

### 2.5. Histological Examination

Freshly isolated adipose tissue of the liver and epididymis was fixed with paraformaldehyde, following which it was dehydrated, paraffin-embedded, and sectioned (5 μm). The processed sections were stained with hematoxylin and eosin and imaged with a light microscope (Leica Instruments Ltd., Wetzlar, Germany).

### 2.6. Gut Microbiome Analysis

Cecal contents DNA of the gut bacteria was extracted from the samples using an E.Z.N.A.^®^ soil DNA kit according to the manufacturer’s instructions. The quality of DNA was evaluated by 1% agarose gel electrophoresis, after purification; the concentration was determined using a NanoDrop spectrophotometer. The extracted genomic DNA was amplified based on the bacterial 16S rRNA gene V3-V4 hypervariable region using conventional barcoded primer pairs 338F (5′-ACTCCTACGGGAGGCAGCAG-3′) and 806R (5′-GGACTACHVGGGTWTCTAAT-3′). Sequencing libraries were generated using a NEXTFLEX Rapid DNA-Seq kit. Sequencing was performed on an Illumina Miseq PE300 platform by Shanghai Majorbio Bio-pharm Technology Co., Ltd. (Shanghai, China). Three replicates were determined.

Microbial α-diversity including Chao, ACE, Shannon, Simpson, and coverage indexes and β-diversity were analyzed by Mothur (V 1.30.2) and Qiime (2020.2.0), respectively, and they were displayed with R software (V 3.3.1). 

### 2.7. Gut Metabolite Analysis

We accurately weighed 50 mg of cecal content samples in a 2 mL centrifuge tube (6 parallel per group) and added a 6 mm diameter grinding bead. Then, 400 µL of extraction solution (methanol: water = 4:1 (*v*:*v*)) containing 0.02 mg/mL of the internal standard (L-2-chlorophenylalanine) was used for metabolite extraction. The Thermo UHPLC-Q Exactive system, used for chromatographic separation of the metabolites, was equipped with an HSS T3 column at a flow rate of 0.4 mL/min. Raw data were processed using Progenesis QI 2.3 to detect and align peaks. The result of the preprocessing produces a data matrix with retention times, mass-to-charge ratios (*m*/*z*), and peak intensities. At least 80% of the metabolomic profiles were retained in any given set of samples. Following the filtration process, for samples where metabolite levels were below the detection threshold, we estimated the values of these minimal metabolites. Subsequently, each metabolic feature was normalized by sum. To maintain data quality control (QC) and evaluate metabolic features, internal standards were employed, and any profiles assessed by QC that had a relative standard deviation (RSD) >30% needed to be removed. Post-normalization and imputation processes, log-transformed data were analyzed statistically to detect significant differences in metabolite concentrations among the groups being compared [16]. Mass spectra of the metabolic features were identified by searching reliable biochemical databases, such as the Human Metabolome Database (HMDB) (http://www.hmdb.ca (accessed on 4 January 2022)) and Metlin database (https://doi.org/metlin.scripps.edu (accessed on 4 January 2022)), and using the accurate mass, MS/MS fragments spectra, and isotope ratio difference. 

The principal component analysis (PCA), partial least squares discriminant analysis (PLS-DA), and orthogonal partial least squares discriminant analysis (OPLS-DA) were performed using ropls package (v 1.6.2) in R. Statistical significance was found when the variables were considered important to predict (VIP) >1 and *p* < 0.05. Significantly different metabolites were screened with a volcano plot based on the *p*-value and fold change (*p* < 0.05, |log_2_fold change (FC)| > 1.0 or 1.2). The KEGG database (Kyoto Encyclopedia of Genes and Genomes, http://www.genome.jp/kegg (accessed on 5 January 2022)) was used to identify the major biochemical pathways and signal transduction pathways enriched in differential metabolites. 

### 2.8. Transcriptome Analysis

Liver samples from the HFD and CLSP_AJ_ groups were selected for transcriptome analysis, 3 parallel per group. Total RNA was extracted from the tissue using TRIzol^®^ Reagent following the manufacturer’s instructions (Invitrogen, Carlsbad, CA, USA), and genomic DNA was removed using DNase I (Takara, San Jose, CA, USA). RNA quality was then assessed using the 2100 Bioanalyzer (Agilent, Santa Clara, CA, USA) and quantified with the ND-2000 (NanoDrop Technologies, Wilmington, DE, USA). The RNA-seq transcriptome library was prepared according to Zou [17]. In this study, a threshold of |log twofold change| ≥ 2 was used for the assessment of the significance of gene expression differences. FDR-corrected *p*-values were applied using the Benjamini/Hochberg method. Subsequently, functional enrichment analysis was performed using the Kyoto Encyclopedia of Genes and Genomes (KEGG) database. The analysis of KEGG pathways was conducted using Goatools (https://doi.org/github.com/tanghaibao/Goatools (accessed on 16 February 2024)). The cloud platform Majorbio (https://doi.org/www.majorbio.com (accessed on 5 January 2022)) was used to analyze the results. 

### 2.9. Statistical Analysis

The experimental data were presented as means ± standard deviation and processed by using the GraphPad Prism 5.01 (GraphPad Software, Boston, MA, USA). The results were subjected to one-way ANOVA analysis or the Kruskal–Wallis H test followed by Tukey’s multiple comparisons test, with *p* < 0.05 considered as statistically significant (* *p* < 0.05, ** *p* < 0.01, *** *p* < 0.001). The Spearman correlation heatmap diagram was constructed with a software package pheatmap in R (version 3.3.1). 

## 3. Results

### 3.1. Chemical Characterization of CLSP_AJ_

CLSP_AJ_ was extracted from *Apostichopus japonicus* cooking liquid, and the sulfate group, uric acid, and protein contents were 22.82 ± 0.01%, 7.95 ± 1.01%, and 20.18 ± 0.13%, respectively. Its monosaccharides consisted of mannose, glucosamine, glucuronic acid, glucose, galactose, arabinose, and fucose in a molar ratio of 3.04:0.77:0.05:1.00:0.16:0.02:0.70, and the relative molecular weight was calculated to be 4.91 kDa. 

In the FT-IR spectrum (Supplementary results, Figure S1), CLSP_AJ_ exhibited absorption peaks at 3399.24 and 2931.39 cm^−1^ due to *O*-H and C-H stretching vibrations, respectively. The sharp peak at 1656.71 cm^−1^ was assigned to the C-*O* stretching vibration. The peaks at 1409.12 cm^−1^ were attributed to the carboxyl group. The N-H vibration was attributed to the absorption peak at 1547.36 cm^−1^. Stretching vibrations of the C-*O* bond were due to the peaks between 1200 and 900 cm^−1^. An absorption peak appeared at 815.65 cm^−1^, which was characteristic of C-2/3 substituted fucose sulfate and C-6 substituted amino galactose if the case of single sulfate substitution was considered, whereas the absorption was also altered if it was considered to be double sulfate substitution. 

The thermal properties of CLSP_AJ_ were measured by differential scanning calorimetry that indicates the heat loss or gain resulting from a sample’s physical or chemical changes [18]. The heat absorption of CLSP_AJ_ started at 108.2 °C (Supplementary results, Figure S2) and reached a maximum of 123.92 °C with an enthalpy of 170.9 J/g, which was related to the evaporation of water. The exotherm started at 249.81 °C and reached its highest point at 274.74 °C with an enthalpy of 52.38 J/g (Supplementary results, Figure S2).

### 3.2. Effect of CLSP_AJ_ on Body and Organ Weight and Lipid Metabolism

**Figure 1:**
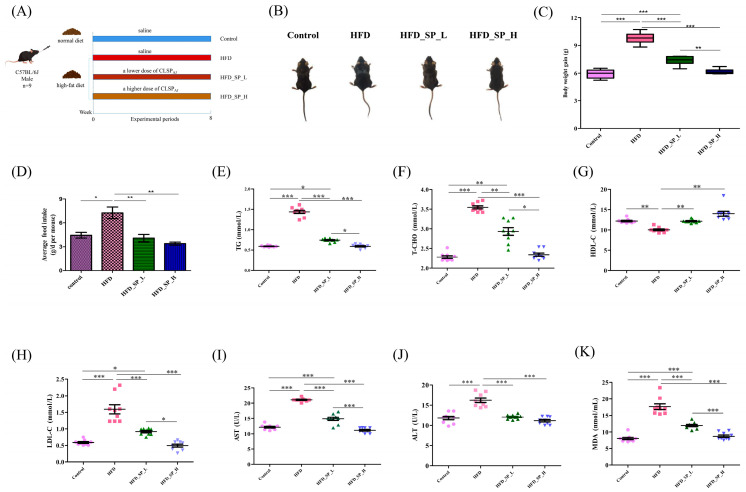
Dietary CLSP_AJ_ improves body weight gain, fat accumulation, and obesity-associated metabolic diseases in HFD-fed mice. (**A**) Study design of in vivo mice experiment, (**B**) average food intake, (**C**) mice morphology, (**D**) body weight gain, (**E**–**K**) serum TG, T-CHO, HDL-c, LDL-c, AST, ALT, and MDA contents. Data are expressed as mean ± SD (n = 9). Graph bars marked by asterisks represent statistically significant differences (*p* < 0.05) based on one-way analysis of variance (ANOVA) with Tukey’s multiple comparisons test. *: *p* < 0.05, **: *p* < 0.01, ***: *p* < 0.001.

To evaluate the impact of CLSP_AJ_ on obesity, mice were subjected to an 8-week HFD with or without the supplementation of CLSP_AJ_ (Figure 1A). The mice in the control group had soft and fine back hair, whereas the mice fed HFD all had greasy, sparse, and lusterless back hair. Both the low-dose and high-dose supplementation groups exhibited significant improvement in the mice, especially in the high-dose group, with finer and more lustrous hair on their backs (Figure 1B). 

Mice fed an HFD had significantly more body weight gain than those fed a standard (maintenance) diet after 8 weeks (*p* < 0.05), whereas a significant decrease was observed for both the HFD_SP_L and HFD_SP_H groups in comparison to the HFD group (*p* < 0.05, Figure 1C). There was indeed a significant difference in total food intake among the HFD-fed groups (Figure 1D), which suggested that the effects of CLSP_AJ_ on body weight might be due to a decrease in food consumption. 

As shown in Figure 1E–H, serum T-CHO, TG, and LDL-C levels in the HFD group were significantly higher than those in the control group, while HDL-C levels were significantly lower. Both the HFD_SP_L and HFD_SP_H groups reduced serum levels of T-CHO, TG, and LDL-C and increased HDL-C levels in obese mice compared to the HFD group. 

ALT and AST levels are used to assess liver cell damage. The HFD led to the increased ALT and AST levels in the liver of obese mice in the HFD group, but oral administration of CLSP_AJ_ reversed these increases and converged to the control group (*p* < 0.05) (Figure 1I,J). 

The free oxygen radicals can attack polyunsaturated fatty acids in biological membranes, thus triggering lipid peroxidation. The level of lipid peroxidation and, indirectly, cellular damage is reflected by the detection of MDA. As shown in Figure 1K, MDA levels were significantly higher in the HFD group compared to the control group, but significantly reduced in groups HFD_SP_L and HFD_SP_H.

**Figure 2:**
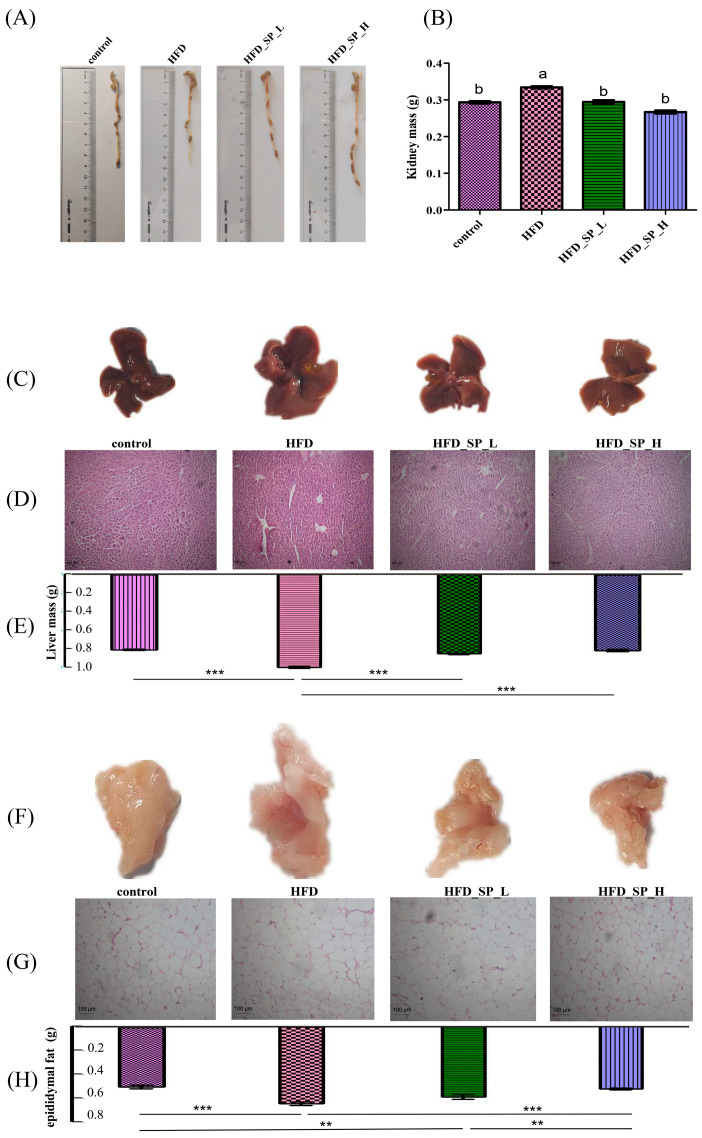
CLSP_AJ_ reduced lipid accumulation and adipocyte enlargement. (**A**) Representative colon length, (**B**) kidney mass, (**C**) liver morphology, (**D**) representative H and E staining images of the liver (200×), (**E**) liver mass, (**F**) epididymal fat morphology, (**G**) representative H and E staining images of the epididymal fat (200×), (**H**) epididymal fat mass. Data are expressed as mean ± SD (n = 9). Graph bars marked by asterisks represent statistically significant differences (*p* < 0.05) based on one-way analysis of variance. Different superscripts (a, b) represent significant differences from each other; *p* < 0.05. **: *p* < 0.01, ***: *p* < 0.001.

The length of the colon was significantly shorter in the HFD group of mice compared to the control group, but it was significantly increased after CLSP_AJ_ intervention (*p* < 0.05) (Figure 2A). 

We also examined whether CLSP_AJ_ could decrease fat and liver hypertrophy. The results were consistent with the effect on body weight, with HFD significantly increasing the amount of liver, kidney, and epididymal fat mass in mice (*p* < 0.05). Compared to the HFD group, the amounts of liver, kidney, and epididymal fat in mice supplemented with different doses of CLSP_AJ_ were significantly reduced in a dose-dependent manner (*p* < 0.05, Figure 2B–H). The results of the histological examination further demonstrated that CLSP_AJ_ intervention significantly alleviated hepatocyte ballooning in HFD-fed mice (Figure 2D). Lipid droplets of epididymal fat were significantly larger in mice in the HFD group compared with the control group, but they were significantly smaller in the low-dose and high-dose supplementation groups, and significantly smaller in the high-dose group than in the low-dose group (Figure 2G).

### 3.3. Effect of CLSP_AJ_ on Gut Microbiota

**Figure 3:**
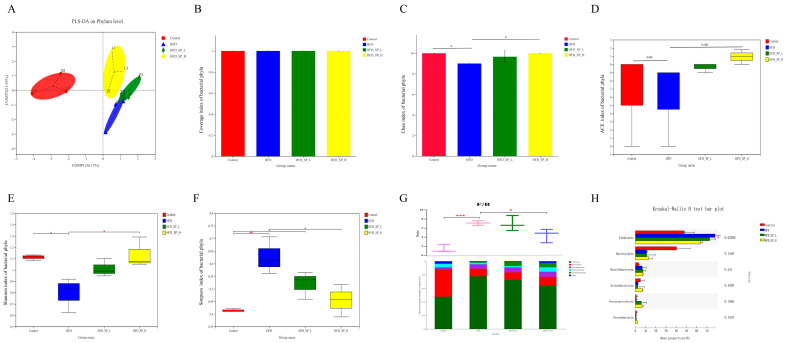
CLSP_AJ_ intervention alters the composition of gut microbiota in HFD-fed mice. (**A**) PLS-DA, (**B**) coverage analysis, (**C**) Chao index, (**D**) ACE index, (**E**) Shannon index, (**F**) Simpson index, (**G**) relative abundance at phylum level and the ratio of Firmicutes to Bacteroidetes, (**H**) Kruskal-Wallis H test analysis at phylum level. Data are expressed as mean ± SD (n = 3). Graph bars marked with ‘*’ in the graph denote statistically significant differences (*p* < 0.05), as determined by one-way analysis of variance (ANOVA) followed by Tukey’s multiple comparison test. *: *p* < 0.05, **: *p* < 0.01, ***: *p* < 0.001.

In our experiment, each treatment group of nine mice was caged in three cages. To make the cecum microbiome more representative, we mixed the cecal contents of mice from each cage evenly and measured them as one sample. A total of 450,509 sequences from 12 cecal content samples were obtained. PLS-DA analysis demonstrated the control, HFD, and HFD_SP_H groups were significantly different, with the HFD_SP_L group showing a transition effect, indicating that CLSP_AJ_ did regulate gut microbiome diversity altered by HFD (Figure 3A). The rarefaction curve and coverage analysis indicated that sequencing data could represent the majority of microbial diversity, characterizing community coverage well (Figure 3B). Consistently, bacterial richness, as indicated by the Chao index (Figure 3C), along with diversity, as demonstrated by the Shannon and Simpson indices, exhibited an increase in mice fed with HFD-fed mice following intervention with CLSP_AJ_ (Figure 3E,F). No significant difference in ACE index was noted (Figure 3D). 

We further analyzed the intestinal microbiota composition at different taxonomic levels in cecal content samples. In mice on a high-fat diet (HFD), there was a higher abundance of the phyla Firmicutes and Desulfobacterota, along with a lower abundance of Bacteroidota and Actinobacteriota, as observed at the phylum level (Figure 3G,H). However, these trends were reversed, and the ratio of Firmicutes to Bacteroidota was reduced (*p* < 0.05) in the HFD-fed mice by CLSP_AJ_ supplementation. 

**Figure 4:**
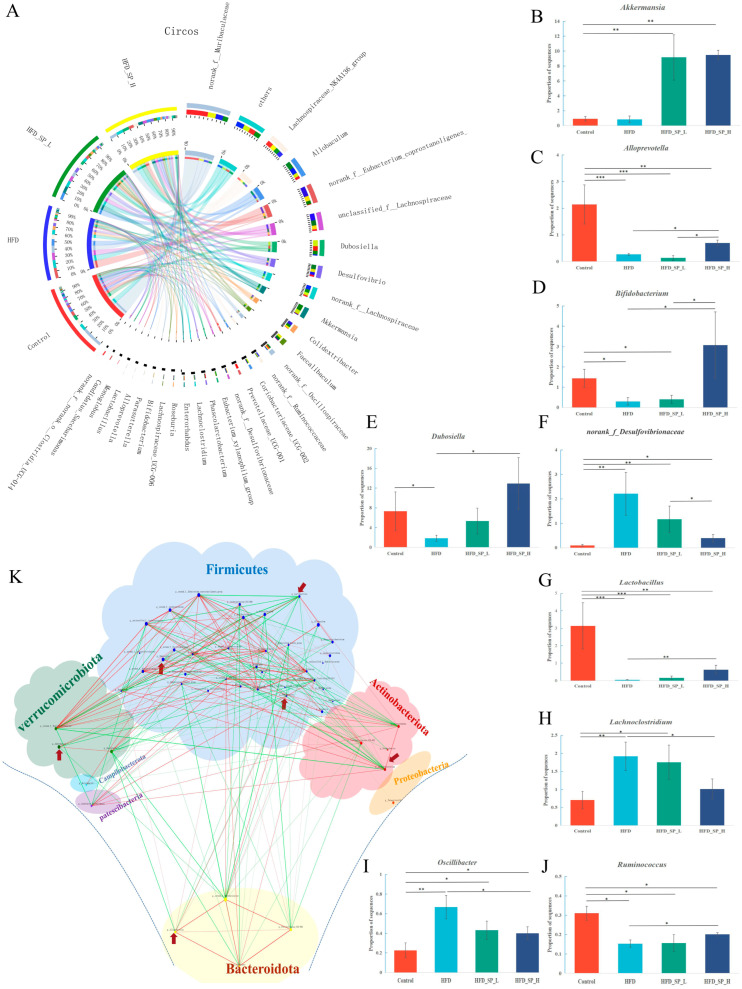
Effects of CLSP_AJ_ on the correlation network diagram and circos analysis at the genus level. (**A**) Circos sample-species relationship diagram. The left part of the inner semicircle represents the species composition in the sample, with the colors of the outer box line representing the subgroup from which it came, the color of the inner box line representing the species, and the length of the color block representing the relative abundance of the species in the corresponding sample. The right part of the outer semicircle represents the proportion of the species distribution in different samples at this taxonomic level, with the outer box line representing the species, the colors of the inner box line representing different subgroups, and the length of the color block representing the distribution of the particular species in the sample. (**B**–**J**) Relative abundances of nine key bacterial genera enriched by CLSP_AJ_. (K) The species relevance network map. The figure displays the leading 50 species characterized by *p* < 0.05 and the absolute correlation coefficient ≥0.5. Node size reflects each species’ relative abundance, with varying colors distinguishing the various species. Red lines denote positive correlations, while green lines signify negative correlations. The thicker the line, the stronger the correlation. Additionally, a greater number of lines between species implies a closer relationship. *: *p* < 0.05, **: *p* < 0.01, ***: *p* < 0.001.

The circos plot showed the correlation between representative genera and different treatment groups (Figure 4A). A comparison was made of the most abundant genera in each group (Figure 4B–J). Despite the increased abundance of potentially pathogenic genera *Lachnoclostridium*, *norank_f_Desulfovibrionaceae,* and *Oscillibacter* in the HFD group, our results suggest that the above trends could be reversed by CLSP_AJ_ supplementation. Moreover, the relative abundances of *Bifidobacterium*, *Alloprevotella*, *Dubosiella*, and *Ruminococcus*, belonging to the beneficial phylotypes, were higher in the CLSP_AJ_ groups than the HFD group. In particular, the intervention with CLSP_AJ_ significantly enhanced the relative abundance of *Lactobacillus* and *Akkermansia*. Collectively, these results indicated that CLSP_AJ_ supplementation can regulate the gut microbiota dysbiosis in HFD-fed mice. 

To explore further the relationship between beneficial phylotypes and other genera, a network analysis was used to highlight the taxa that might be inter-related. We found that the beneficial phylotypes were located at the denser part of the network map (as the arrows point in Figure 4K); the degrees of *Bifidobacterium*, *Lactobacillus*, *Akkermansia*, *Alloprevotella*, *Dubosiella*, and *Ruminococcus* were 16, 16, 8, 10, 7, and 9, respectively, and their clustering coefficients were 0.79, 0.70, 0.57, 0.51, 0.71, and 0.36, respectively. These findings also corroborated that the CLSP_AJ_ supplementation improved the gut microbiota in HFD-fed mice.

### 3.4. Effects of CLSP_AJ_ on Alterations of the Metabolic Pathway

**Figure 5:**
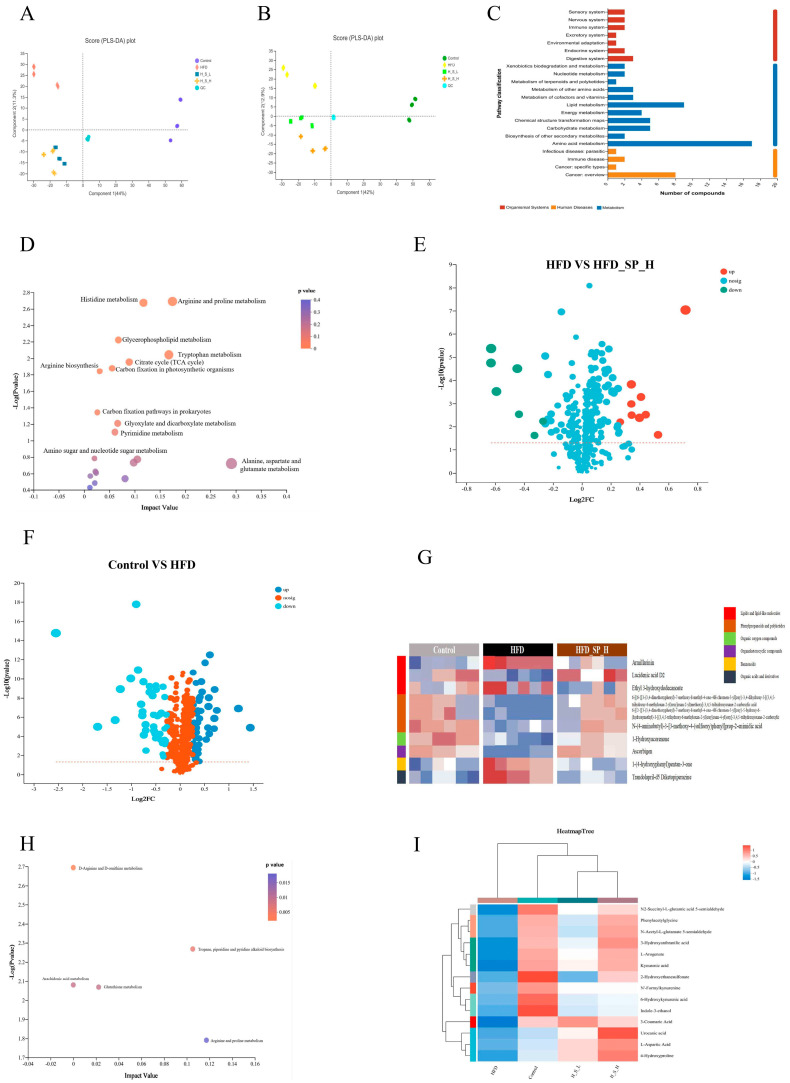
CLSP_AJ_ supplementation modifies gut metabolite levels in cecal content samples (n = 6). PLS-DA score plot among the four experimental groups in (**A**) cationic and (**B**) anionic mode. (**C**) Differences in KEGG functional pathways between HFD and HFD_SP_H. (**D**) CLSP_AJ_ regulates the metabolic pathways in HFD-induced mice. (**E**,**F**) Volcano plots of altered metabolites with *p* < 0.05 and |log_2_FC| > 1.2 between HFD and HFD_SP_H (**E**) and control and HFD (**F**). The size and color of each circle were based on the pathway impact value and the *p*-value. (**G**) CLSP_AJ_ regulates the metabolic pathways in HFD-induced mice with *p* < 0.05 and |log_2_FC| > 1.2 between HFD and HFD_SP_H. (**H**) Heatmap of the top 16 differential metabolites between HFD and HFD_SP_H. (**I**) Heatmap of the metabolites in the control group altered by HFD responding to HFD_SP_H treatment; colored from blue for low levels to red for high levels.

A peak alignment of the metabolomics data revealed that a comprehensive sum of 569 metabolites were identified, of which 305 and 264 metabolites were identified in the positive and negative ion modes, respectively. The results of the PLS-DA score plot to assess data quality and identify underlying biomarkers showed clear separations among the metabolic profiles (Figure 5A,B), indicating that the metabolites of cecal contents differed significantly among the experimental groups. 

Given that the effect of HFD_SP_H was greater than that of HFD_SP_L supplementation in HFD-fed mice, to clarify further the effect of CLSP_AJ_ supplementation on metabolites associated with obesity in mice, data from the HFD_SP_H group were utilized for subsequent analysis. OPLS-DA analysis revealed a clear separation of the mouse groups into distinct clusters (Supplementary results, Figure S4), confirming that different dietary patterns and CLSP_AJ_ supplementations led to differences in metabolites. In cationic and anionic modes, 78 and 72 different metabolites were found in the HFD_SP_H and HFD groups, respectively. The HFD_SP_H treatment resulted in upregulation of 51 and 45 and down-regulation of 27 and 27 differential metabolites in the positive and negative ion mode, respectively, compared to the HFD group (Supplementary results, Tables S1 and S2; VIP > 1, *p* < 0.05). 

The metabolite expression patterns in samples from the HFD and HFD_SP_H groups were differentiated by PLS-DA as shown in Figure 5A,B. Then, KEGG functional analysis of the differential pathways was performed. Among the organismal systems, the differential pathways were attributed mainly to the digestive system, and were concentrated mainly in amino acid metabolism, being linked predominantly to cancer and to a lesser extent to immune diseases (Figure 5C). 

Then, we further investigated the metabolic pathways associated with the differential metabolites (VIP > 1 and *p* < 0.05). The data showed that CLSP_AJ_ affected mainly the metabolism of alanine, aspartate, glutamate, arginine and proline, histidine, and tryptophan (Figure 5D).

The above metabolites were further screened using the threshold values of *p* < 0.05 and |log_2_FC| > 1.2, and 16 metabolites were obtained as candidate indicators for subsequent analysis (Figure 5E). The metabolites altered by the supplementation of CLSP_AJ_ at the high dose included lipids and lipid-like molecules (5), phenylpropanoids and polyketides (4), organoheterocyclic compounds (2), organic oxygen compounds (2), benzenoids (1), organic acids and derivatives (1), and organic nitrogen compounds (1). When comparing the control and HFD groups (Figure 5F), 89 altered metabolites were found to be triggered by the HFD feeding (*p* < 0.05 and |log_2_FC| > 1.2), but notably, the metabolites changed upon HFD feeding were partially restored after the CLSP_AJ_ supplementation (Figure 5G). Specifically, CLSP_AJ_ reversed the reduction of lucidenic acid D2, 6-[(6-{[3-(3,4-dimethoxyphenyl)-7-methoxy-8-methyl-4-oxo-4H-chromen-5-yl]oxy} -3,4-dihydroxy-5-[(3,4,5-trihydroxy-6-methyloxan-2-yl)oxy]oxan-2-yl)methoxy]-3,4,5-trihydroxyoxane-2-carboxylic acid (6_6_XX), 6-[(2-{[3-(3,4-dimethoxyphenyl)-7-methoxy-8-methyl-4-oxo-4H-chromen-5-yl]oxy}-5-hydroxy-6-(hydroxymethyl)-3-[(3,4,5-trihydroxy-6-methyloxan-2-yl)oxy]oxan-4-yl)oxy]-3,4,5-trihydroxyoxane-2-carboxylic acid (6_2_YY), N-(4-aminobutyl)-3-[3-methoxy-4-(sulfooxy)phenyl]prop-2-enimidic acid (N_4_aminobutyl_ZZ), 1-hydroxyacorenone, and ascorbigen, and reversed the increase in armillarinin, ethyl-3-hydroxydodecanoate, 1-(4-hydroxyphenyl)pentan-3-one, and trandolapril-d5 diketopiperazine caused by HFD. Then, we also investigated the relevant metabolic pathways, and the data indicated that the arginine and proline metabolism, tropane, piperidine, and pyridine alkaloid biosynthesis, D-arginine and D-ornithine metabolism, arachidonic acid metabolism, and glutathione metabolism were key pathways between HFD and HFD_SP_H groups (Figure 5H). 

Amino acid metabolites were analyzed by clustering, and the results showed that CLSP_AJ_ restored the metabolite changes caused by HFD to some extent, especially N2-succinyl-L-glutamic acid 5-semialdehyde, phenylacetylglycine, N-acetyl-L-glutamate 5-semialdehyde, 3-hydroxyanthranilic acid, L-arogenate, kynurenic acid, 2-hydroxyethanesulfonate, and 3-coumaric acid (Figure 5I). 

### 3.5. CLSP_AJ_ Regulated Liver Transcriptome

**Figure 6:**
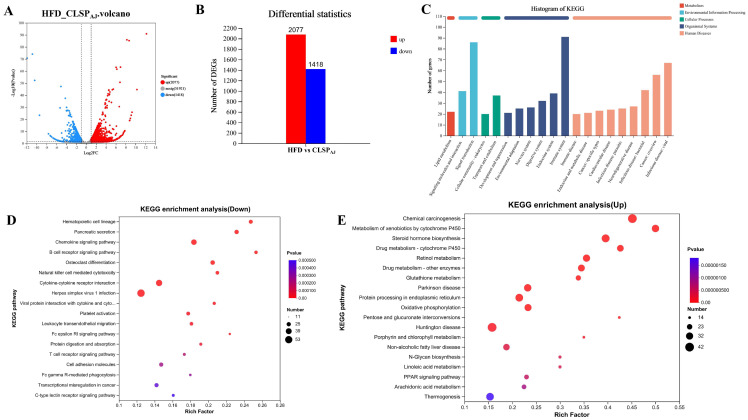
Analysis of transcriptome between CLSP_AJ_ and HFD groups. (**A**) Volcano map for gene expression differences. (**B**) Statistical histograms of up-regulated and down-regulated genes. (**C**) Functional annotation of DEGs. (**D**) Enrichment analysis of KEGG pathways in down-regulated Genes. (**E**) Enrichment analysis of KEGG pathways in up-regulated genes. KEGG, Kyoto Encyclopedia of Genes and Genomes; HFD, high-fat diet; CLSP_AJ_, sulfated polysaccharides from *Apostichopus japonicus* cooking liquid.

To gain a deeper understanding of the weight loss effect of CLSP_AJ_ intervention on HFD mice, transcriptome analysis was conducted on the liver tissues from both the HFD and CLSP_AJ_ groups. In total, 3495 differentially expressed genes (DEGs) have been identified, of which 2077 are up-regulated and 1418 down-regulated (Figure 6A,B). Based on the classification of KEGG pathways, one pathway was associated with metabolism, two with environmental information processing, two with cellular processes, six with organismal systems, and nine with human diseases pathways (Figure 6C). The KEGG enrichment results also revealed that down-regulated genes were primarily linked to the metabolism of the B cell receptor signaling pathway, hematopoietic cell lineage, and pancreatic secretion (*p* < 0.001, Figure 6E). The genes that were up-regulated primarily showed an enrichment in xenobiotic metabolism via cytochrome P450, chemical carcinogenesis, and drug metabolism—cytochrome P450 (*p* < 0.001, Figure 6D).

### 3.6. Microbiome, Metabolome and Transcriptome Correlation Analysis

#### 3.6.1. Correlation Analysis of Obesity-Related indices with Microbiome and Metabolite Profiles

**Figure 7:**
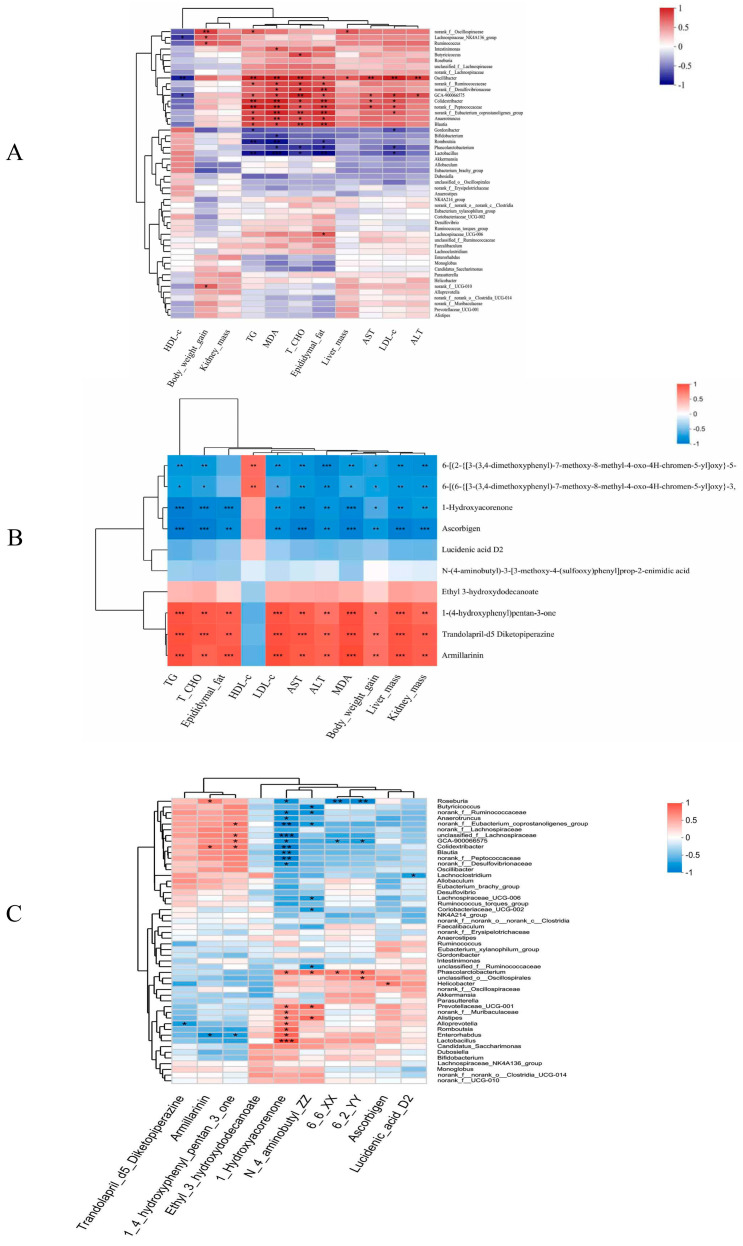
Analysis of the association between gut microbiome and metabolites with obesity-related indices following CLSP_AJ_ supplementation. (**A**) Analysis of the Spearman’s correlation between the gut microbiome and indices related to obesity. (**B**) Analysis of the Spearman’s correlation between the gut metabolites and indices related to obesity. (**C**) Analysis of the Spearman’s correlation between the obesity-related gut microbiome and metabolites. The different colors indicate different correlations: blue denotes a negative correlation, whereas red signifies a positive one. Correlations of significance are highlighted with asterisks. *: *p* < 0.05, **: *p* < 0.01, ***: *p* < 0.001.

We performed a correlation analysis of the altered microbiome with indices of adiposity (body weight gain, kidney mass, liver mass, epididymal fat mass, colon length, serum TG, T-CHO, HDL-c, LDL-c, AST, ALT, and MDA). Among microbial taxa, *Gordonibacter*, *Bifidobacterium*, *Romboutsia*, *Phascolarctobacterium*, *Lactobacillus*, *Akkermansia*, *Allobaculum*, and *Eubacterium_brachy_group* were negatively linked with the obesity-related indices (Figure 7A), indicating their potential role in obesity prevention. By contrast, *Oscillibacter*, *norank_f__Ruminococcaceae*, *norank_f__Desulfovibrionaceae*, *GCA-900066575*, *Colidextribacter*, *norank_f__Peptococcaceae*, *norank_f__Eubacterium_coprostanoligenes_group*, *Anaerotruncus*, and *Blautia* were significantly and positively correlated with obesity-related indices (Figure 7A), suggesting that they may have an important role in the pathogenesis of obesity development. 

In addition, we discovered that 6_2_YY, 6_6_XX, 1-hydroxyacorenone, ascorbigen, and lucidenic acid D2 may play a crucial role in preventing obesity, whereas 1-(4-hydroxyphenyl) pentan-3-one, trandolapril-d5 diketopiperazine, and armillarinin may play a vital role in the process of obesity development (Figure 7B).

#### 3.6.2. CLSP_AJ_ Altered the Gut Microbiome and Related Metabolite Profiles in HFD-Fed Mice

We identified an intrinsic link between changes in the intestinal microbiome and related metabolites with the anti-obesity effect of CLSP_AJ_.

In order to elucidate the relationship between gut microbiota and metabolites, a correlation analysis was carried out focusing on the 50 most abundant genera (based on community heatmap analysis results) and the metabolites after the CLSP_AJ_ supplementation (*p* < 0.05 and |log_2_FC| > 1.2) (Figure 7C). A significant negative correlation was observed between the 1-hydroxyacorenone (organic oxygen compounds) (decreased by CLSP_AJ_) and the relative abundance of *Roseburia*, *norank_f__Eubacterium_coprostanoligenes_group*, *unclassified_f__Lachnospiraceae*, *GCA-900066575*, *Colidextribacter, norank_f__Ruminococcaceae*, *Anaerotruncus*, *Blautia*, *norank_f__Peptococcaceae*, and *norank_f__Desulfovibrionaceae*, and was positively correlated with *Phascolarctobacterium*, *Prevotellaceae_UCG-001*, *norank_f__Muribaculaceae*, *Alistipes*, *Alloprevotella*, *Romboutsia*, *Enterorhabdus*, and *Lactobacillus*. The important intestinal keystone bacterium genus *Roseburia* was significantly negatively correlated with two phenylpropanoids and polyketides (6_6_XX and 6_2_YY) and was positively correlated with armillarinin (lipids and lipid-like molecules). The relative abundance of core intestinal genus *Phascolarctobacterium* showed a significant positive correlation with three phenylpropanoids and polyketides (N_4_aminobutyl_ZZ, 6_6_XX, and 6_2_YY).

**Figure 8:**
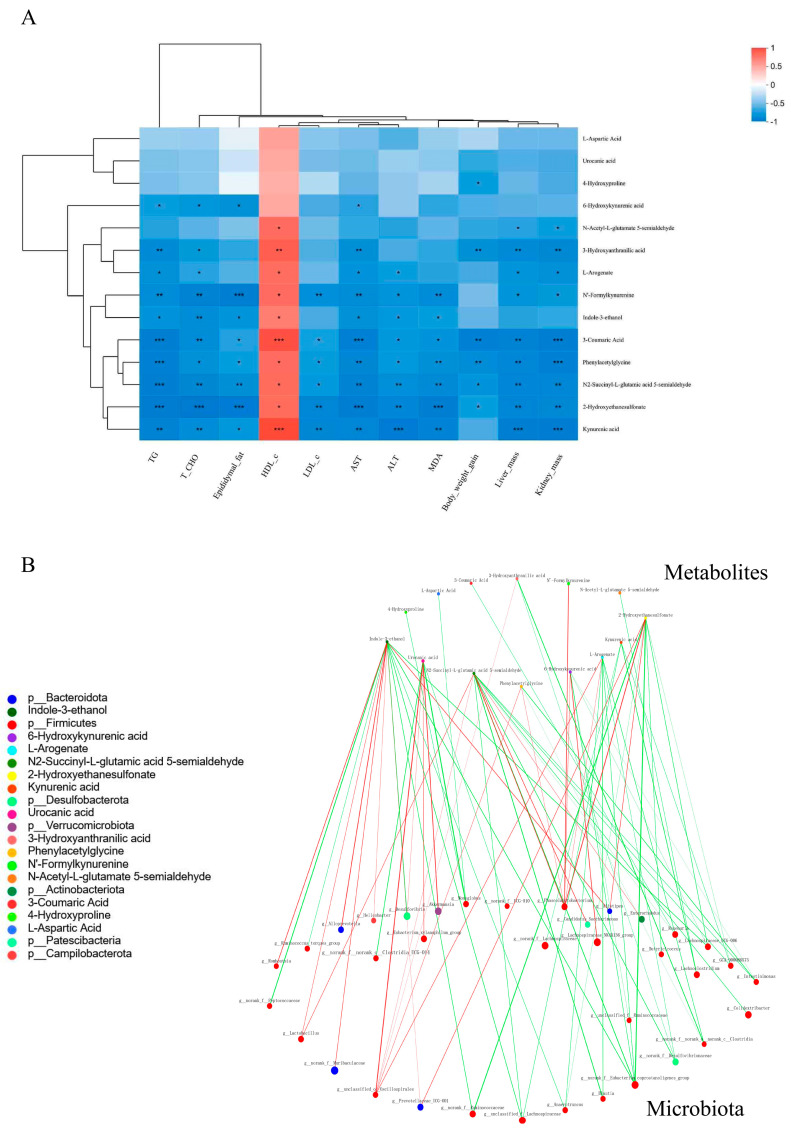
Analysis of the association between gut microbiome and amino acid metabolites with obesity-related phenotypes following CLSP_AJ_ supplementation. (**A**) Analysis of the Spearman’s correlation between the amino acid metabolites and phenotypes related to obesity. The different colors indicate different correlations: blue denotes a negative correlation, whereas red signifies a positive one. Correlations of significance are highlighted with asterisks. *: *p* < 0.05, **: *p* < 0.01, ***: *p* < 0.001. (**B**) Correlation networks showing associations between the obesity-related gut microbiome and amino acid metabolites. The strength of the correlation is represented proportionately by the width and color of the edges, with red indicating a positive correlation and green indicating a negative one. Only strong (Spearman |r| ≥ 0.5) and significant (*p* < 0.05) correlations were displayed.

Because differential metabolites were enriched mainly in amino acid metabolism, the Spearman’s rank correlation analysis of altered amino acid metabolites (AAM) and obesity-related indices was performed to identify potential associations. All the AAM were correlated positively with HDL-c and negatively with other indices (Figure 8A). 

We conducted an in-depth analysis of the anti-obesity mechanism associated with CLSP_AJ_ by analyzing the correlation network between gut bacteria and AAM. The analysis suggested a potential correlation between gut microbial taxa associated with obesity and multiple AAM (Figure 8B, *p* < 0.05). It is worth noting that indole-3-ethanol, N2-succinyl-L-glutamic acid 5-semialdehyde, urocanic acid, 2-hydroxyethanesulfonate, and L-arogenate were located in very important positions of the network and were closely related to a variety of genera, e.g., *Akkermansia*, *Lactobacillus*, *Roseburia*, and *Phascolarctobacterium*. Taken together, these results indicated that changes in the relative abundance of key microbial taxa in the gut and corresponding changes in metabolites were the major contributors to the anti-obesity effects of CLSP_AJ_ supplementation.

#### 3.6.3. Microbiome–Transcriptome Correlation Analysis

Microbiome–transcriptome correlation analyses were performed to identify the relationship between gut microbiota and genes. Using Spearman’s correlation coefficient, we performed an analysis of gut microbiota at the genus level in conjunction with genes related to obesity-enriched pathways. These pathways included amino acid metabolism, xenobiotics by cytochrome P450, NF-Kappa B signaling, FoxO signaling pathway, Jak-STAT signaling pathway, cytokine–cytokine receptor interaction, PI3K-Akt signaling pathway, MAPK signaling pathway, Rap1 signaling pathway, cGMP-PKG signaling pathway, and intestinal immune network for IgA production. 

**Figure 9:**
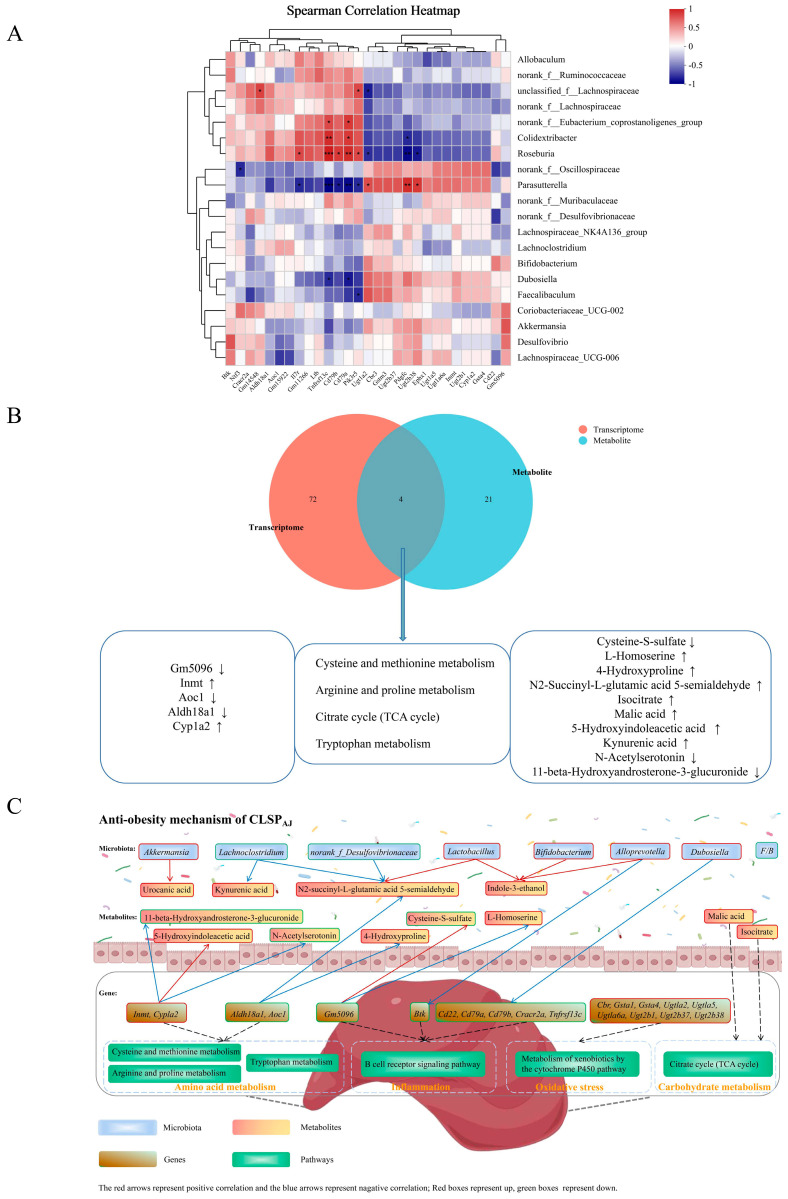
Correlation analysis among the microbiome, metabolome, and transcriptome. (**A**) Spearman’s correlation between genus-level gut microbiota and differential genes in obesity-related pathways. (**B**) Conjoint analysis of the metabolome and transcriptome. (**C**) Schematic of CLSP_AJ_ resistance to the mechanism of high-fat-induced obesity. The strength of the correlation is represented proportionately by the width and color of the edges, with red indicating a positive correlation and blue indicating a negative one. *: *p* < 0.05, **: *p* < 0.01, ***: *p* < 0.001.

As shown in Figure 9A, unclassified_f__Lachnospiraceae, Dubosiella, norank_f__Eubacterium_coprostanoligenes_group, Colidextribacter, Roseburia, Parasutterella, and Faecalibaculum were significantly enriched with obesity-related KEGG pathways. Faecalibaculum had a significant negative correlation with Pik3r5. Dubosiella showed a significant negative correlation with Tnfrsf13c and Cd79a, norank_f__Eubacterium_coprostanoligenes_group, in contrast to Dubosiella. Unclassified_f__Lachnospiraceae had a significantly positive correlation with Gm14548 and Pik3r5, but a negative correlation with Ugt1a2. Colidextribacter was significantly positively correlated with Tnfrsf13c and Cd79a but negatively correlated with Pdgfc. Roseburia was significantly positively correlated with Il7r, Tnfrsf13c, Cd79b, Cd79a, and Pik3r5 but negatively correlated with Ugt1a2, Pdgfc, and Ugt2b38, in contrast to Parasutterella. 

#### 3.6.4. Metabolome–Transcriptome Correlation Analysis

We performed a comprehensive analysis of the transcriptome and metabolome with the expectation of determining the relationship between DEGs and metabolites. A total of 72 KEGG pathways were changed at the transcriptional level, whereas 21 exhibited changes at the metabolome level, as depicted in Figure 9B. Among them, four KEGG pathways were found to co-exist in both the transcriptome and metabolome datasets. These pathways included cysteine and methionine metabolism, arginine and proline metabolism, and tryptophan metabolism, all belonging to amino acid metabolism. Additionally, citrate cycle (TCA cycle) was categorized under carbohydrate metabolism.

## 4. Discussion

Fat deposition and adipocyte hypertrophy in the liver result from the energy imbalance created by obesity [19,20]. In the present study, a HFD increased obesity, and the CLSP_AJ_ supplementation significantly ameliorated this change. The body, organ, and fat weights showed the same trend in each treatment group. Furthermore, histopathology revealed a significant accumulation of lipids in the liver tissues of mice that were fed a high-fat diet (HFD), and serum AST, ALT, TC, TG, and LDL-C decreased significantly after CLSP_AJ_ supplementation, indicating that CLSP_AJ_ was effective in alleviating liver injury caused by a HFD and the potential anti-obesity ability [21]. Following TG accumulation in the liver, overactive fatty acid metabolism escalates the formation of reactive oxygen species, precipitating oxidative stress [22]. MDA is a lipid peroxidation product that reflects oxidative damage in hepatocytes. In the present research, CLSP_AJ_ reduced oxidative stress as evidenced by significantly decreased MDA levels in the HFD group. All these results could be an indication that the dietary supplementation of CLSP_AJ_ was effective in suppressing HFD-induced obesity. 

Dietary polysaccharides can act as a carbon source for gut microbiome, promote the growth of beneficial bacteria, and exert their prebiotic effects by modulating the composition of gut microbiota and the metabolite production, thereby inhibiting the development of obesity and related diseases [14,23,24]. The results of this study showed that the supplementation with CLSP_AJ_ significantly increased gut microbial diversity in HFD-fed mice, thus suggesting that CLSP_AJ_ could reverse intestinal disorders and promote a healthy intestine. 

The increase in the Firmicutes/Bacteroidetes ratio has been associated with obesity, with Firmicutes more abundant in obese people, and Bacteroidetes more abundant in lean individuals [25,26]. In the work presented here, CLSP_AJ_ reduced the HFD-induced increase in the relative abundance of Firmicutes and greatly reduced the Firmicutes/Bacteroidota ratio, suggesting that CLSP_AJ_ regulation of the gut microbiome contributes to the reduction of HFD-induced obesity. 

The HFD altered the abundance of gut bacteria at the genus level. Relevant research on the regulation of intestinal microflora found that a HFD can alter the abundance at the genera and lead to a shift in the dominant bacterial taxa from Bacteroideae to Lachnospiraceae and Akkermansiaceae to *Eubacterium* [27,28]. In our present work, we also noticed an increase in the abundance of *unclassified_f_Lachnospiraceae* and *norank_f_Eubacterium_coprostanoligenes_group* and a decrease in *Akkermansia* in the HFD group. We then performed a Kruskal–Wallis analysis to examine the differences among the treatments and found that CLSP_AJ_ intervention significantly increased the abundance of beneficial bacteria *Akkermansia*, *Bifidobacterium*, *Alloprevotella*, *Lactobacillus*, and *Ruminococcus*. *Akkermansia* is commonly found in the human digestive tract. *Akkermansia muciniphila* is an intestinal commensal bacterium that has great potential as a next-generation probiotic to influence the intestinal microbiota indirectly, creating conditions for the production of “beneficial metabolites” and enhancing “beneficial bacteria” [29], supporting intestinal homeostasis and organismal health, and has the potential to inhibit obesity and the metabolic disorders it triggers [30]. Several studies have found that *Bifidobacterium* and *Lactobacillus* are relatively safe probiotics in humans, whereby an increase in the relative abundance of *Bifidobacterium* spp. can help maintain intestinal integrity and reduce the accumulation of harmful bacteria and metabolic endotoxins [31]. In particular, *Bifidobacterium* spp. was found to reduce inflammatory factors and endotoxins by repairing and regulating T and B lymphocytes in high-fat-diet mice. Some other studies have shown that *Bifidobacterium* also has an important role in the control of body fat content [32]. We found that *Alloprevotella*, *Lactobacillus,* and *Ruminococcus* could also suppress HFD-induced obesity [33,34,35]. Correlation analysis further showed that these genera played a positive role in improving obesity-related indices. Importantly, we reached the same conclusions by the species-related network analysis. Taken together, the obesity-suppressing properties of CLSP_AJ_ were likely due to altering the relative abundance of specific bacteria. 

The most significant differential metabolic pathway between the HFD groups without and with CLSP_AJ_ supplementation was amino acid metabolism. The variations in amino acid composition and abundance affect the structure and function of the gut microorganisms, thereby regulating the gut microbiota immune axis as well as other signaling pathways that are critically important in regulating the intestinal flora and contribute to intestinal environmental homeostasis [36]. Our correlation analysis results also confirmed that amino acid metabolites played a positive role in improving obesity-related indices. Amino acids with branched chains along with aromatic amino acids are key factors in lipid metabolism disorders such as obesity [37]. These amino acids in turn can generate a wide range of metabolic by-products such as SCFA, branched-chain fatty acids, and ammonia [26]. We observed that CLSP_AJ_ supplementation, the HFD-induced indole-3-ethanol, N2-succinyl-L-glutamic acid 5-semialdehyde, and urocanic acid in mice (belonging to tryptophan metabolism, arginine, and proline metabolism, and histidine metabolism, respectively) were significantly increased; these metabolites may play a crucial role in the prevention of obesity. 

The metabolic processing of amino acids by bacteria produces biogenic amines, including cadaverine and agmatine, which arise from the decarboxylation of lysine and arginine, respectively. Agmatine, by elevating cAMP levels in tissues, has the ability to emulate the weight reduction outcomes of calorie limitation. Biogenic amines have far-reaching physiological effects in vivo, e.g., *Lactobacillus plantarum* makes the decarboxylation of ornithine to putrescine, increasing its fraction in the urine of obese individuals; therefore, patients with obesity-related hypertension have lower arterial blood pressure and body mass index. These showed a positive correlation with increased counts of Lactobacillus [38]. Thus, microbially mediated intestinal amino acid metabolism is involved in the lipid metabolism of the host.

Dietary polysaccharides could have their anti-obesity effects through gut microbiome-driven metabolites. We found that indole-3-ethanol, N2-succinyl-L-glutamic acid 5-semialdehyde, and urocanic acid were at the center of the correlation network and interacted with the relative abundance of *Akkermansia*, *Lactobacillus*, *Roseburia*, *Phascolarctobacterium*, and other genera. *Akkermansia* and *Lactobacillus* have been shown to suppress the development of obesity caused by HFD, which we also demonstrated in this study. *Roseburia intestinalis*, a common butyric acid-producing bacterium (phylum Firmicutes), is one of the key bacteria in the human intestine for the degradation of dietary fiber xylan and, together with other xylan-degrading bacteria, can be important in the utilization of dietary polysaccharides [39]. *Phascolarctobacterium* produces SCFAs and may colonize the human gastrointestinal tract in large quantities, which may be potentially linked to the metabolic state and mood of the host [40]. In individuals more prone to weight loss, the intestine has a higher relative abundance of *Phascolarctobacterium* spp. [41]. In addition to weight loss, *Phascolarctobacterium* is also a key regulator of the dynamic balance of the gut microbiota [42]. Therefore, the positive effects of indole-3-ethanol, N2-succinyl-L-glutamic acid 5-semialdehyde, and urocanic acid in ameliorating HFD-induced inflammation, and as potential mediators of CLSP_AJ_-facilitated anti-obesity effects on the gut microbiomee, warrant further research.

Transcriptomic data indicated that the B cell receptor signaling pathway was significantly down-regulated by CLSP_AJ_, indicating that CLSP_AJ_ could ameliorate HFD-induced obesity through the mediation of inflammatory pathways. The B cell receptor signaling pathway biomarkers are *Cd22*, *Cd79a*, *Cd79b*, and *Cracr2a*. *CD79* is a heterodimeric molecule involved in signal transduction in components of the B cell receptor, consisting of two peptide chains, *CD79a* and *CD79b*, which are highly specific to the B cell lineage [43]. *CD22*, mainly expressed in mature B cells, is a cell surface adhesion molecule with a role in regulating B cell activation and contributing to the control of B cell sensitivity to antigenic responses [44]. *Cracr2a* is expressed in T cells and regulates cell signaling and calcium in-flow, and has been implicated in human disease in genome-wide correlation studies [45]. After the intervention with CLSP_AJ_, the primary metabolic pathway that is up-regulated involves the metabolism of xenobiotics by cytochrome P450, involving *Gstm3*, *Cbr3*, *Gsta1*, *Gsta4*, *Ugt1a2*, *Ugt1a5*, *Ugt1a6a*, *Ugt2b1*, *Ugt2b37*, and *Ugt2b38*, with *Gstm3*, *Gsta1*, and *Gsta4* also associated with glutathione metabolism. Oxidative stress is one of the factors involved in the pathogenesis of obesity, and increased expression of *Gsta* and *Gstm3* has been shown to reduce the levels of oxidative stress associated with obesity [46]. Furthermore, Nrf2/ARE signaling, an important anti-oxidative stress mechanism, is impaired in obesity. Nrf2 activation is protective of metabolic health, and *cbr3* is one of the genes characterizing Nrf2. It has been shown that the knockout of *Nrf2* in mice has a significantly reduced *Cbr3* expression in the liver [47], suggesting that *cbr3* is positively correlated with Nrf2, i.e., involved in metabolic health. Uridine diphosphate glucuronosyltransferase (UGT) is one of the most important enzymes in human phase II metabolism and is involved in the metabolic clearance of many drugs. UDP glucuronosyltransferases (UGTs) are one of the most important enzymes in human phase II metabolism, and their signaling is an important determinant of bile acid metabolic homeostasis, which can be involved in the metabolic clearance of many drugs by regulating the bile acid homeostasis acting downstream of FXR-FGF15 signaling [48]. It has been shown that the expression and activity of phase II enzymes (*Ugt1a*, Ugt2b, Nat, and *Gstt*) are decreased in the liver of obese rats [49]. Thus, the down-regulation of oxidative stress-related genes within the cytochrome P450 pathway during the metabolism of xenobiotics could potentially explain the protective effects of CLSP_AJ_ against obesity in mice.

Combined gut flora and DEGs analysis was performed and the results indicated that *Dubosiella* and *Parasutterella* were significantly negatively correlated with genes in the B cell receptor signaling pathway. It has been demonstrated that *Dubosiella* and *Parasutterella* are negatively correlated with inflammation [50,51]. Thus, they may modulate the inflammatory response via the B cell receptor signaling pathway, thereby reducing obesity.

Combined DEGs and differential metabolites were visualized in integrated analyses at the transcriptome and metabolome pathway levels. The co-analysis of KEGG pathways revealed that ten differential metabolites (Cysteine-S-sulfate, L-Homoserine, 4-Hydroxyproline, N2-succinyl-L-glutamic acid 5-semialdehyde, isocitrate, malic acid, 5-Hydroxyindoleacetic acid, kynurenic acid, N-Acetylserotonin, and 11-beta-Hydroxyandrosterone-3-glucuronide) and five DEGs (*Gm5096*, *Inmt*, *Aoc1*, *Aldh18a1*, and *Cyp1a2*) were enriched in four obesity-related pathways. Of the ten metabolites mentioned above, isocitrate and malic acid are involved in the carbohydrate pathway, while the other eight are associated with amino acid metabolism. *Inmt*, a gene involved in tryptophan metabolism, is one of the most prominent genes associated with inhibitory regulation, attention, and executive function, and has been implicated in studies linking obesity to cognitive decline [52]. The *AOC1* is an important target for the treatment of autoimmune and inflammatory diseases due to its role in immune cell migration, e.g., for the intervention of ulcerative colitis [53]. Obesity-induced complications and metabolic disorders contribute to the development of hepatocellular carcinoma. It has been found that the proline biosynthesis pathway contributes to the growth of hepatocellular carcinoma tumors, and *Aldh18a1* is one of the targets of the proline biosynthesis pathway, promoting the proliferation of hepatocellular carcinoma cells [54]. Caffeine’s ability to aid in weight loss and reduce the risk of obesity-related diseases has been linked to *Cyp1a2* and *Ahr* (which regulates the expression of *Cyp1a2*) associated with the rate of caffeine metabolism [55]. The integrated analyses of transcriptomic and metabolomic showed and confirmed that CLSP_AJ_ primarily prevents obesity by regulating amino acid metabolism.

## 5. Conclusions

CLSP_AJ_, especially at the high dose, ameliorated HFD-induced obesity, as demonstrated by a reduction in body weight, hepatic lipid accumulation, adipocyte hypertrophy, and a decrease in serum lipid levels in HFD mice. Subsequent studies on the microbiome, transcriptome, and metabolomics revealed that CLSP_AJ_ favorably influenced structural and functional alterations in the intestinal flora, promoting an increased abundance of beneficial bacteria, which mediated changes in various genes and metabolites. The gut microbiota was associated with genes and metabolites in obesity-linked pathways, including carbohydrate metabolism and the most significant metabolic pathway being amino acid metabolism. All of the analyses revealed that CLSP_AJ_ had a good regulatory impact on obesogenic metabolic disorders. Therefore, as a by-product of sea cucumber processing, CLSP_AJ_ may be an effective candidate for functional anti-obesity products.

## Data Availability

The original contributions presented in the study are included in the article/Supplementary Material, further inquiries can be directed to the corresponding author.

## Supplementary Materials

The following supporting information can be downloaded at: https://www.mdpi.com/article/10.3390/foods13132017/s1, Figure S1: FT-IR spectrum of CLSP_AJ_; Figure S2: Thermodynamic spectrum of CLSP_AJ_; Figure S3: Rarefaction curves; Figure S4: Cluster analysis of metabolites among groups (OPLS-DA score plots); Table S1: Significantly named metabolites between the HFD_SP_H and HFD groups were identified in the positive ion mode; Table S2: Significantly named metabolites between the HFD_SP_H and HFD groups were identified in the negative ion mode.
