# Supplementary material for: Sulfated Polysaccharides from Sea Cucumber Cooking Liquid Prevents Obesity by Modulating Gut Microbiome, Transcriptome, and Metabolite Profiles in Mice Fed a High-Fat Diet
Source: Foods. 2024 Jun 26;13(13):2017. doi: 10.3390/foods13132017 (PMC11241695; doi:10.3390/foods13132017)
Supplement: Supplementary file 1 [file foods-13-02017-s001.zip › foods-3059957-supplementary.pdf]

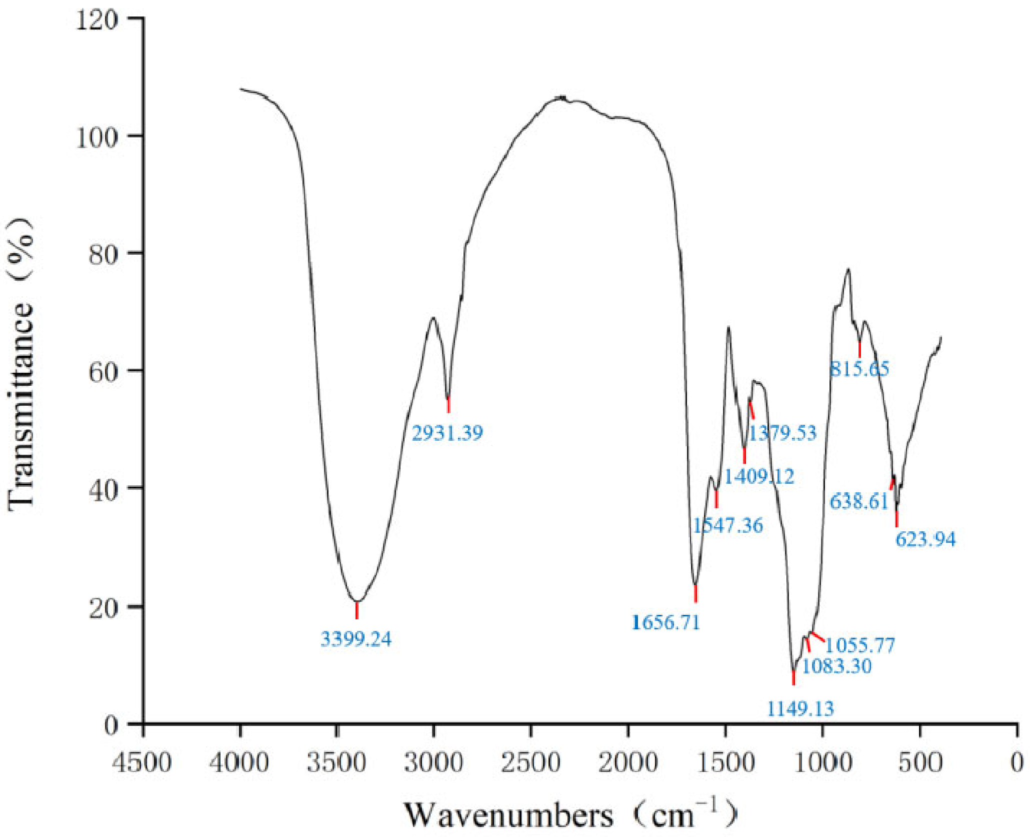

Fig. S1 FT-IR spectrum of $CLSP_{AJ}$

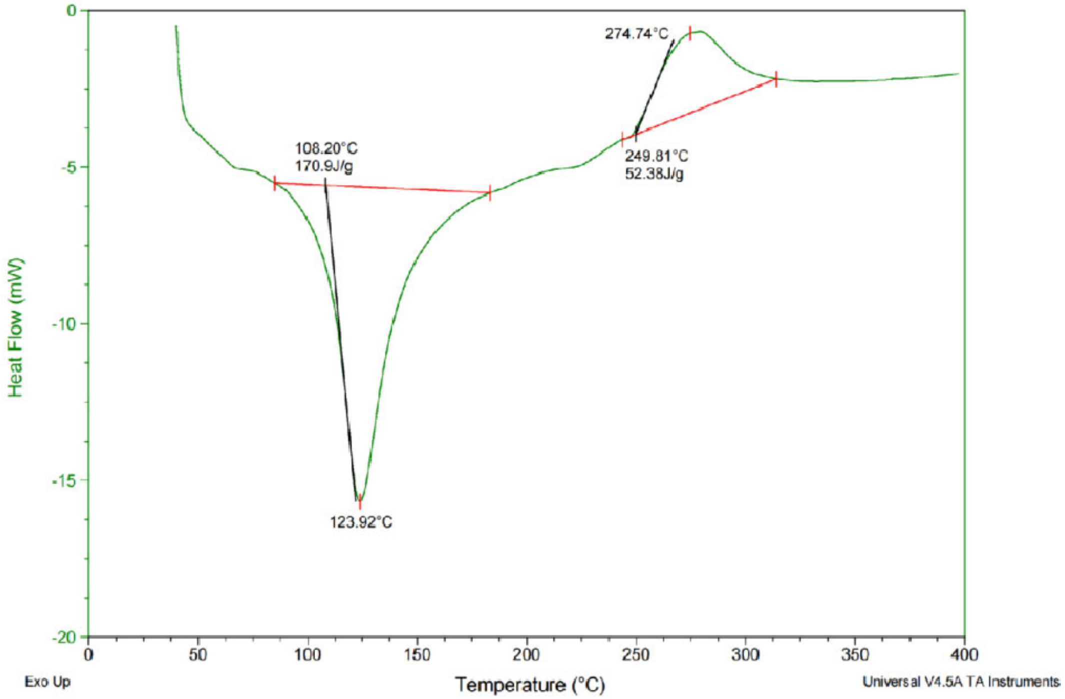

Fig. S2 Thermodynamic spectrum of $CLSP_{AJ}$

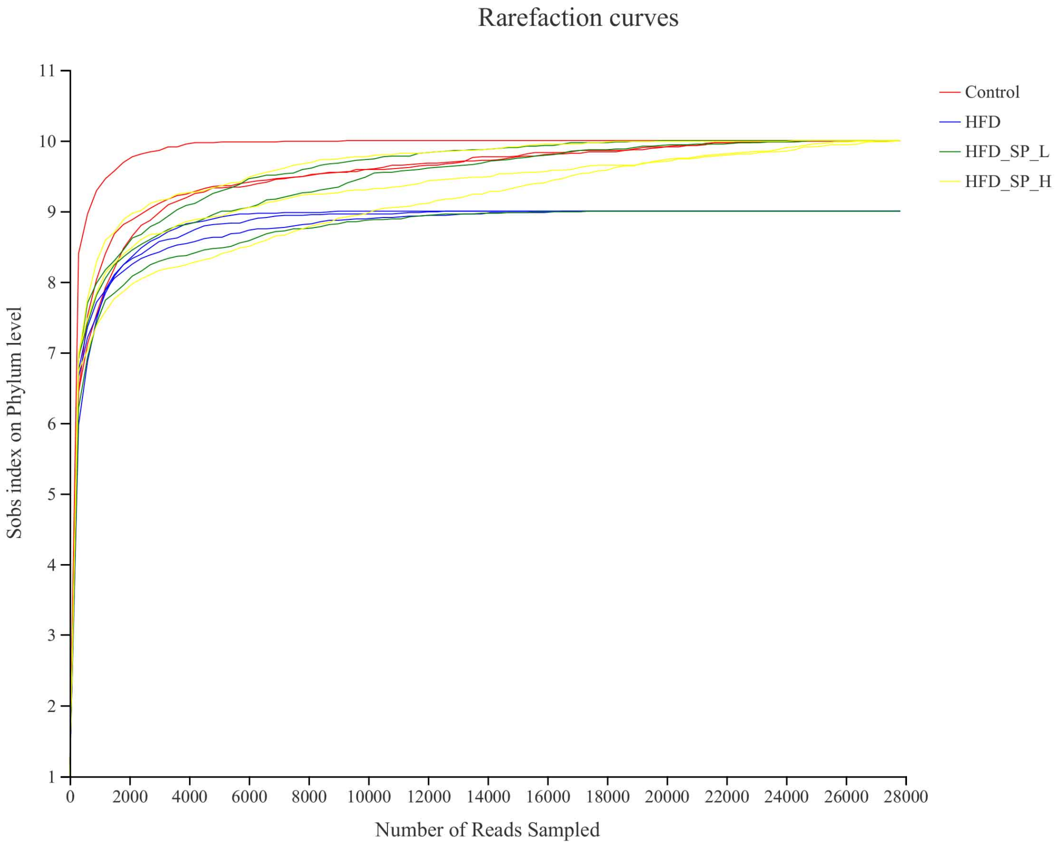

Fig. S3 Rarefaction curves

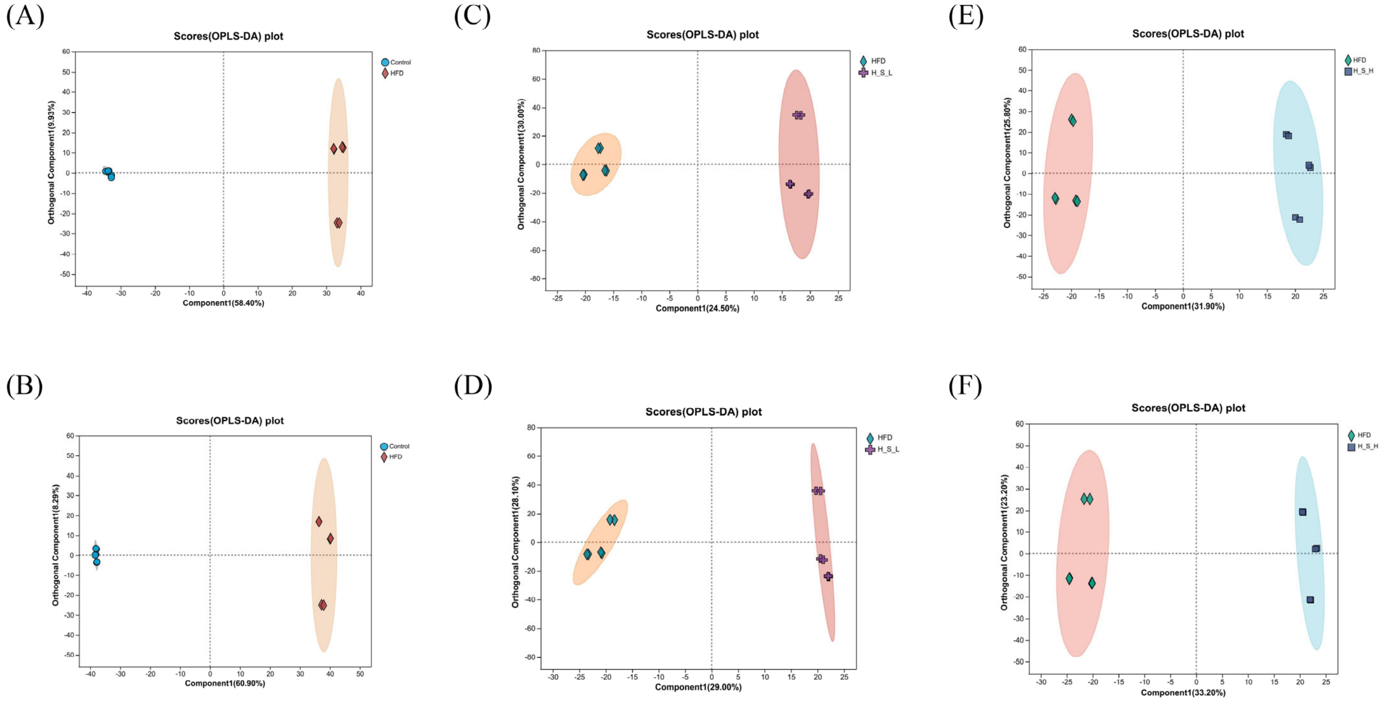

Fig. S4 Cluster analysis of metabolites among groups (OPLS-DA score plots).

(A) HFD vs Control, positive ion mode, R2X = 0.584, R2Y = 0.969, Q2 = 0.955; (B) HFD vs Control, negative ion mode, R2X = 0.609, R2Y = 0.984, Q2 = 0.973; (C) HFD_SP_L vs HFD, positive ion mode, R2X = 0.245, R2Y = 0.838, Q2 = 0.679; (D) HFD_SP_L vs HFD, negative ion mode, R2X = 0.29, R2Y = 0.949, Q2 = 0.847; (E) HFD_SP_H vs HFD, positive ion mode, R2X = 0.319, R2Y = 0.925, Q2 = 0.809 and (F) HFD_SP_H vs HFD, negative ion mode, R2X = 0.332, R2Y = 0.96, Q2 = 0.868.

Table S1 Significantly named metabolites between the HFD_SP_H and HFD groups were identified in the positive ion mode.

ID	Metabolite	Regulate	M/Z	Retention time	Formula	Fragmentation Score	Theoretical Fragmentation Score	Mass Error	KEGG Pathway Description	VIP_pred_OPLS-DA	VIP_PLS-DA	FC (H_S_H/HFD)	*P*_value	FDR
pos_26	N-Methyl-L-glutamate	up	144.0655	0.773683333	C6H11NO4	59.4	-	-0.2613	Methane metabolism; Metabolic pathways; Microbial metabolism in diverse environments	1.0081	0.9741	1.0269	0.0009	0.0092
pos_124	4-Hydroxyproline galactoside	up	276.1075	1.601833333	C11H19NO8	-	55.4	-0.8458	-	1.3227	1.3155	1.0542	0.0028	0.0180
pos_224	16R-HETE	down	353.2680	6.667933333	C20H32O3	64.6	-	-1.1695	Arachidonic acid metabolism	1.2210	1.2091	0.9594	0.0032	0.0196
pos_309	N-Acetyl-a-neuraminic acid	up	310.1128	0.545883333	C11H19NO9	84	-	-1.3745	-	1.2883	1.2487	1.0491	0.0022	0.0158
pos_510	Serylserine	up	175.0713	0.5528	C6H12N2O5	-	60.2	-0.3419	-	1.0143	1.0326	1.0500	0.0253	0.0679

pos_515	N-Acetyl-L-glutamate 5-semialdehyde	up	138.0548	0.559716667	C7H11NO4	-	52.6	-0.6218	Metabolic pathways; Arginine biosynthesis; Biosynthesis of secondary metabolites; 2-Oxocarboxylic acid metabolism; Arginine and proline metabolism; Biosynthesis of amino acids	1.2289	1.1969	1.0461	0.0003	0.0050
pos_530	7-Hydroxyticlopidine	up	302.0374	0.566733333	C14H14ClNOS	-	70.5	-0.9045	-	1.9259	1.8007	1.1196	0.0002	0.0040
pos_800	Zedoarondiol	up	235.1691	5.6107	C15H24O3	-	68.6	-0.5041	-	1.9050	1.8803	1.1548	0.0003	0.0049
pos_946	ID14326	down	526.2925	6.7168	C28H36N4O3S	-	55	6.0945	-	1.4265	1.3767	0.9411	0.0090	0.0348
pos_952	PE(18:2/0:0)	down	478.2926	6.779633333	C23H44NO7P	52.5	-	-0.3589	-	1.4101	1.4068	0.9564	0.0001	0.0023
pos_955	PC(20:4/0:0)	down	544.3395	6.793633333	C28H50NO7P	80.6	-	-0.5023	-	1.3063	1.3043	0.9576	0.0091	0.0350
pos_1004	PE(P-18:0/0:0)	up	466.3291	7.128166667	C23H48NO6P	58.3	-	-0.3247	-	1.4042	1.3861	1.0477	0.0000	0.0005
pos_1247	12-hydroxyheptadecanoic acid	down	309.2420	6.793633333	C17H34O3	-	58.7	6.9886	-	1.3419	1.2587	0.9402	0.0245	0.0663
pos_1291	3alpha,7alpha-Dihydroxycoprostanic	up	417.3360	6.639966667	C27H46O4	-	54.4	-0.7369	-	1.1714	1.1524	1.0381	0.0004	0.0051

	acid													
pos_1301	(3beta,5alpha,9alpha,22E,24R)-3,5,9-Trihydroxy-23-methylergosta-7,22-dien-6-one	down	522.3551	6.6121	C29H46O4	-	78.3	-0.6082	-	1.0576	1.0767	0.9693	0.0274	0.0718
pos_1399	3-Hydroxy-L-proline	up	164.0917	0.71855	C5H9NO3	-	56.6	0.3825	-	1.2004	1.1391	1.0497	0.0054	0.0261
pos_1603	Urocanic acid	up	139.0501	1.276366667	C6H6N2O2	92.4	-	1.0498	Metabolic pathways; Histidine metabolism	1.2797	1.2488	1.0363	0.0002	0.0035
pos_2096	Diacetone alcohol	up	134.1175	0.573633333	C6H12O2	-	64.6	-0.0628	-	1.6887	1.7728	1.1101	0.0049	0.0249
pos_2105	N-Acetylgalactosamine	up	465.1688	0.573633333	C8H15NO6	-	65	-0.6663	Amoebiasis	1.5148	1.4269	1.0612	0.0001	0.0033
pos_2128	Kinetin-7-N-glucoside	up	422.1054	0.59455	C16H19N5O6	-	68.8	1.9836	-	1.2076	1.1160	1.0482	0.0024	0.0167
pos_2211	Glycerophosphocholine	down	258.1093	0.711716667	C8H20NO6P	57.6	-	-2.9417	Ether lipid metabolism; Glycerophospholipid metabolism; Choline metabolism in cancer	1.1635	1.1225	0.9579	0.0018	0.0139

pos_2245	6-Feruloylglucose 2,3,4-trihydroxy-3-methylbutylglycoside	up	457.1658	0.746216667	C21H30O12	-	62.7	-9.6893	-	1.1852	1.1429	1.0456	0.0036	0.0209
pos_2304	Beta-D-Galactopyranosyl-(1->4)-2-amino-2-deoxy-beta-D-glucopyranosyl-(1->6)-D-mannose	up	486.1812	0.808566667	C18H33NO15	-	51	-1.1003	-	1.5216	1.4759	1.0972	0.0281	0.0733
pos_2339	N-Acetylcadaverine	down	145.1335	0.850566667	C7H16N2O	63.9	-	-0.3747	-	1.7190	1.6407	0.8842	0.0147	0.0471
pos_2780	L-Arogenate	up	228.0865	2.806733333	C10H13NO5	62.2	-	-0.4659	Metabolic pathways; Phenylalanine, tyrosine and tryptophan biosynthesis; Biosynthesis of secondary metabolites; Biosynthesis of amino acids	1.7222	1.7132	1.1323	0.0062	0.0282
pos_2796	N6,N6-Dimethyladenosine	up	296.1346	2.848433333	C12H17N5O4	72.4	-	-2.4063	-	2.0853	2.0415	1.1589	0.0001	0.0024
pos_2828	Phenylalanylproline	up	295.1649	2.946966667	C14H18N2O3	-	56.2	-1.1950	-	1.0297	1.0649	1.0516	0.0266	0.0705

pos_2850	3,4,5-trihydroxy-6-(3,4,5-trihydroxycyclohex-1-ene-1-carbonyloxy)oxane-2-carboxylic acid	up	368.1197	3.038466667	C13H18O11	-	59.1	2.8109	-	2.2635	2.2493	1.1889	0.0002	0.0043
pos_2869	4-Amino-4-deoxychorismic acid	up	226.0709	3.102233333	C10H11NO5	60.2	-	-0.2325	-	1.3152	1.2663	1.0578	0.0010	0.0100
pos_2908	N-Acetylserotonin	down	219.1127	3.20765	C12H14N2O2	59.6	-	-0.7024	Metabolic pathways; Tryptophan metabolism	2.2488	2.1631	0.8384	0.0000	0.0007
pos_2964	Hydroxyprolyl-Lysine	up	323.1704	3.395666667	C11H21N3O4	-	57.4	5.4013	-	1.2402	1.2528	1.0797	0.0433	0.1001
pos_3007	L-phenylalanyl-L-proline	down	263.1388	3.54385	C14H18N2O3	88	-	-0.9333	-	1.1876	1.2149	0.9581	0.0039	0.0219
pos_3110	3,4,5-trihydroxy-6-{4-hydroxy-3-[2-(3-hydroxy-5-methoxyphenyl)ethyl]phenoxy}oxane-2-carboxylic acid	down	469.1702	4.016233333	C21H24O10	-	61.7	-0.4884	-	3.2289	3.1656	0.6463	0.0000	0.0011
pos_3121	(2E,4E)-2,7-Dimethyl-2,4-octadienedioic acid	up	240.1229	4.08625	C10H14O4	-	82.9	-0.7030	-	1.1779	1.1563	1.0577	0.0004	0.0056
pos_3143	Methyl (3b,11x)-3-Hydroxy-8-oxo-6-eremoph	up	322.2010	4.206583333	C16H24O4	-	53.4	-0.8861	-	1.7113	1.7218	1.1918	0.0198	0.0575

	ilen-12-oate														
pos_3309	Corey PG-Lactone Diol	up	269.1745	5.288016667	C15H24 O4	64.7	-	-0.8301	-	1.8934	1.8662	1.1549	0.0003	0.0044
pos_3320	Trandolapril-d5 Diketopiperazine	down	430.2693	5.344016667	C24H32 N2O4	-	58.6	-1.6969	-	3.3512	3.2617	0.7334	0.0000	0.0014
pos_3390	LysoPE(0:0/18:2(9 Z,12Z))	down	460.2801	5.793233333	C23H44 NO7P	-	72.2	-4.4990	-	2.0604	2.0538	0.8624	0.0029	0.0182
pos_3408	Indole-3-ethanol	up	162.0913	5.898883333	C10H11 NO	86.4	-	-0.4749	Tryptophan metabolism	1.5098	1.4030	1.0908	0.0059	0.0273
pos_3421	Zanthodioline	up	347.1598	5.9479	C16H19 NO5	-	70.3	-1.2277	-	1.2203	1.1992	1.0427	0.0000	0.0006
pos_3492	Nivalenol	up	351.0860	6.115883333	C15H20 O7	-	51.4	6.1360	Sesquiterpenoid and triterpenoid biosynthesis	1.8313	1.8509	1.1955	0.0169	0.0514
pos_3498	LysoPE(0:0/16:1(9 Z))	up	515.2823	6.122866667	C21H42 NO7P	-	55.1	-7.5428	-	1.1064	1.0440	1.0411	0.0088	0.0344
pos_3574	Ingol	up	367.2104	6.26975	C20H30 O6	51.3	-	-2.9859	-	1.4942	1.4304	1.0684	0.0008	0.0085
pos_3718	PC(20:3/0:0)	down	546.3552	6.535266667	C28H52 NO7P	77.6	-	-0.3967	-	1.1905	1.1346	0.9618	0.0235	0.0644
pos_3753	(+/-)13-Azaprostan oic acid	up	312.2893	6.591116667	C19H37 NO2	51.7	-	-1.3538	-	1.4962	1.4744	1.0900	0.0094	0.0358
pos_3938	PC(18:1/0:0)	down	522.3551	6.995983333	C26H52 NO7P	79.5	-	-0.6515	-	1.1515	1.1664	0.9655	0.0212	0.0604

pos_4058	PE(P-20:0/0:0)	up	494.3600	7.3312	C25H52 NO6P	51.2	-	-1.0528	-	1.4047	1.4249	1.0760	0.0025	0.0170
pos_5159	(3beta,22E,24R)-3-Hydroxyergosta-5, 8,22-trien-7-one	up	393.3151	7.219183333	C28H42 O2	-	78.9	-0.1898	-	1.5484	1.5433	1.0814	0.0004	0.0053
pos_5254	PC(20:2/0:0)	down	548.3705	7.0375	C28H54 NO7P	76.2	-	-1.1108	-	1.1918	1.1539	0.9633	0.0032	0.0196
pos_5307	PC(22:4/0:0)	down	572.3705	6.954333333	C30H54 NO7P	55.7	-	-0.9646	-	1.5696	1.5336	0.9188	0.0153	0.0482
pos_5394	LysoPC(22:5(4Z,7 Z,10Z,13Z,16Z))	down	570.3545	6.849483333	C30H52 NO7P	57.4	-	-1.5518	Glycerophospholipid metabolism; Choline metabolism in cancer	1.5066	1.4856	0.9415	0.0015	0.0126
pos_5406	7,8-Dihydrovomifo liol 9-[rhamnosyl-(1-> 6)-glucoside]	up	567.2969	6.83555	C25H42 O12	-	76.3	-7.8712	-	2.8918	2.7741	1.3182	0.0042	0.0228
pos_5419	Lucidenic acid D2	up	537.2494	6.807633333	C29H38 O8	-	76.5	6.8061	-	2.9556	2.8418	1.2678	0.0002	0.0034
pos_5422	Phosphocholine	down	184.0731	6.807633333	C5H14N O4P	85.2	-	-1.0370	Metabolic pathways; Glycerophospholipid metabolism; Choline metabolism in cancer	1.5458	1.4928	0.9351	0.0026	0.0172
pos_5470	PC(22:6/0:0)	down	568.3394	6.74475	C30H50	58.3	-	-0.6866	-	1.4416	1.4477	0.9483	0.0061	0.0279

					NO7P									
pos_5484	(Z)-7-Hexadecen-1,16-olide	down	285.2420	6.7238	C16H28O2	-	50.2	-1.5400	-	1.0109	1.0659	0.9624	0.0094	0.0356
pos_5569	D-Urobilin	up	611.2859	6.639966667	C33H40N4O6	-	76.5	3.2211	Metabolic pathways; Biosynthesis of secondary metabolites; Porphyrin and chlorophyll metabolism	1.7951	1.7327	1.0923	0.0005	0.0063
pos_5747	Biliverdin	up	583.2546	6.458383333	C33H34N4O6	-	53.9	-0.8062	Metabolic pathways; Biosynthesis of secondary metabolites; Porphyrin and chlorophyll metabolism	1.6389	1.5834	1.0662	0.0000	0.0014
pos_5917	LysoSM(d18:1)	down	507.3793	6.304733333	C23H50N2O5P+	-	55.4	-0.5435	Sphingolipid metabolism	1.0968	1.0147	0.9523	0.0481	0.1079
pos_6065	7-Hydroxytrichodermol	up	267.1587	6.115883333	C15H22O4	51.6	-	-1.3207	-	1.3256	1.3082	1.0586	0.0005	0.0063
pos_6110	Ethyl 3-hydroxydodecanoate	down	262.2375	6.0529	C14H28O3	-	62.9	-0.7352	-	2.1195	2.1584	0.8300	0.0059	0.0274
pos_6122	1-(4-hydroxyphenyl)pentan-3-one	down	220.1330	6.0249	C11H14O2	-	63.9	-0.9592	-	3.5830	3.5163	0.6468	0.0000	0.0004
pos_6145	Kynurenic acid	up	190.0498	5.961916667	C10H7NO3	54.5	-	-0.5817	Metabolic pathways; Tryptophan metabolism	1.7534	1.7465	1.1185	0.0001	0.0019

pos_6324	6-[3,4-dihydroxy-6-(hydroxymethyl)-5-methoxyoxan-2-yl]-5,7-dihydroxy-2-(4-hydroxy-3-methoxyphenyl)-4H-chromen-4-one	up	441.1175	5.176016667	C23H24O11	-	71.9	-1.0135	-	2.1133	2.0472	1.1658	0.0004	0.0051
pos_6408	3-Coumaric Acid	up	147.0440	4.762966667	C9H8O3	75.2	-	-0.4271	Metabolic pathways; Degradation of aromatic compounds; Phenylalanine metabolism; Microbial metabolism in diverse environments	1.6030	1.5388	1.0836	0.0001	0.0024
pos_6513	13-Hydroxy-5'-O-methylmelledonal	down	480.2227	4.213583333	C24H30O9	-	52	-0.3169	-	2.0495	2.0743	0.8388	0.0077	0.0320
pos_6542	Carboxyprimaquine	up	275.1387	4.1079	C15H18N2O3	61.9	-	-0.9967	-	1.3905	1.4653	1.1006	0.0085	0.0338
pos_6549	Riboflavin (Vitamin B2)	up	377.1452	4.065233333	C17H20N4O6	62	-	-1.0580	-	1.5894	1.5544	1.0827	0.0003	0.0044
pos_6551	L-Pyridosine	up	277.1181	4.05825	C12H18N2O4	-	78	8.6825	-	1.3030	1.3574	1.0957	0.0178	0.0534
pos_6909	Succinylproline	up	216.0866	2.995966667	C9H13NO5	84.5	-	-0.1640	Renin-angiotensin system inhibitors	1.0550	1.0466	1.0398	0.0005	0.0064

pos_6926	Succinoadenosine	up	384.1147	2.946966667	C14H17 N5O8	50.2	-	-0.8785	-	1.3736	1.3542	1.0608	0.0116	0.0406
pos_7213	3-Hydroxyanthranilic acid	up	154.0498	2.281383333	C7H7NO3	85.2	-	-0.4860	Metabolic pathways; Aminobenzoate degradation; Tryptophan metabolism; Microbial metabolism in diverse environments	1.0658	1.0198	1.0342	0.0003	0.0045
pos_7441	Cis-4-Hydroxycyclohexylacetic acid	up	176.1280	1.503983333	C8H14O3	-	56.5	-0.5911	-	1.9153	1.9419	1.1844	0.0109	0.0394

pos_8252	Putrescine	down	89.1078	0.580583333	C4H12N2	77	-	5.3347	Metabolic pathways; Glutathione metabolism; Biosynthesis of secondary metabolites; D-Arginine and D-ornithine metabolism; ABC transporters; Arginine and proline metabolism; Protein digestion and absorption; Tropane, piperidine and pyridine alkaloid biosynthesis; Biosynthesis of alkaloids derived from ornithine, lysine and nicotinic acid; Biosynthesis of plant secondary metabolites	2.1048	2.0006	0.7964	0.0240	0.0656

pos_8576	Histamine	down	112.0872	0.442216667	C5H9N3	83.5	-	2.3241	Metabolic pathways; Asthma; Fc epsilon RI signaling pathway; Biosynthesis of secondary metabolites; Inflammatory mediator regulation of TRP channels; Histidine metabolism; Gastric acid secretion; Protein digestion and absorption; Histamine H2/H3 receptor agonists/antagonists; Neuroactive ligand-receptor interaction; Biosynthesis of alkaloids derived from histidine and purine; Synaptic vesicle cycle	1.6648	1.5437	0.8904	0.0012	0.0113
pos_494	4-Hydroxyproline	up	164.0917	0.53205	C5H9NO3	-	59.6	0.2593	Metabolic pathways; ABC transporters; Arginine and proline metabolism	1.3748	1.3308	1.0681	0.0015	0.0129

pos_6687	6-Hydroxykynurenic acid	up	206.0447	3.57185	C10H7NO4	60.4	-	-0.2570	Tryptophan metabolism	1.0518	0.9476	1.0436	0.0355	0.0868
pos_123	N-Acetylneuraminic Acid	up	274.0918	0.760066667	C11H19NO9	64.3	-	-0.9879	Metabolic pathways; Amino sugar and nucleotide sugar metabolism	1.0559	1.0088	1.0291	0.0057	0.0271

Table S2 Significantly named metabolites between the HFD_SP_H and HFD groups were identified in the negative ion mode.

ID	Metabolite	Regulate	M/Z	Retention time	Formula	Fragmentation Score	Theoretical Fragmentation Score	Mass Error	KEGG Pathway Description	VIP_pred_OPLS-DA	VIP_PLS-DA	FC (H_S_H/HFD)	*P*_value	FDR
neg_145	2'-Deoxyuridine	up	227.0669	2.263083333	C9H12N2O5	52.5	-	-1.2173	Pyrimidine metabolism; Metabolic pathways; ABC transporters	1.0110	1.0878	1.0515	0.0367	0.0956
neg_491	Methylmalonic acid	down	117.0184	1.788433333	C4H6O4	54.6	-	-8.5017	Pyrimidine metabolism; Propanoate metabolism; Metabolic pathways; Valine, leucine and isoleucine degradation	1.1538	1.1230	0.9659	0.0015	0.0097
neg_536	N2-Succinyl-L-glutamic acid 5-semialdehyde	up	212.0560	2.269983333	C9H13NO6	-	52.3	-2.0777	Metabolic pathways; Arginine and proline metabolism	1.2003	1.1832	1.0467	0.0000	0.0009
neg_625	Succinylacetone	up	203.0556	2.246016667	C7H10O4	-	59.6	-4.3221	-	1.6803	1.6576	1.0998	0.0000	0.0003
neg_627	6-(4-ethyl-2-hydroxyphenoxy)-3,4,5-trihydroxyoxane-2-carboxylic acid	up	351.0569	0.772283333	C14H18O8	-	77.9	6.1681	-	1.4530	1.4817	1.0721	0.0005	0.0047

neg_799	L-Aspartic Acid	up	132.0293	0.517283333	C4H7NO4	86.6	-	-7.0942	Biosynthesis of various secondary metabolites - part 3; Cysteine and methionine metabolism; Carbon metabolism; Alanine, aspartate and glutamate metabolism; Protein digestion and absorption; beta-Alanine metabolism; Bacterial chemotaxis; Pantothenate and CoA biosynthesis; ABC transporters; Carbon fixation in photosynthetic organisms; Neuroactive ligand-receptor interaction; Biosynthesis of alkaloids derived from ornithine, lysine and nicotinic acid; Biosynthesis of plant secondary metabolites; Metabolic pathways; Arginine biosynthesis; Lysine biosynthesis; Microbial metabolism in	1.1229	1.1431	1.0382	0.0004	0.0044

			diverse environments; 2-Oxocarboxylic acid metabolism; Histidine metabolism; Biosynthesis of amino acids; Glycine, serine and threonine metabolism; Monobactam biosynthesis; Biosynthesis of secondary metabolites; Nicotinate and nicotinamide metabolism; Cyanoamino acid metabolism; Two-component system; Aminoacyl-tRNA biosynthesis; Central carbon metabolism in cancer; Biosynthesis of plant hormones

neg_858	(+/-)-Enterolactone	up	297.1130	6.3494	C18H18 O4	84	-	-0.773 5	-	1.9414	1.9853	1.1877	0.0089	0.0346
neg_864	1-Hydroxyacoreno ne	up	271.1339	6.376833333	C15H22 O3	-	54.1	9.1798	-	2.4267	2.4103	1.2676	0.0011	0.0075
neg_901	Xi-7-Hydroxyhexa decanedioic acid	up	323.1862	6.44535	C16H30 O5	-	59.2	-0.838 6	-	1.2278	1.2082	1.0712	0.0400	0.1024
neg_905	Isokobusone	up	267.1600	6.5272	C14H22 O2	-	63.4	-0.913 7	-	1.8620	1.8461	1.1350	0.0002	0.0023
neg_919	12alpha-hydroxy-3 -oxo-5beta-cholan-24-oic Acid	up	389.2695	6.568016667	C24H38 O4	-	51.6	-0.515 0	-	1.0168	1.0258	1.0244	0.0005	0.0047
neg_243 9	2-Methylguanosine	up	296.0997	2.7671	C11H15 N5O5	66.3	-	-1.251 7	-	1.1718	1.1591	1.0477	0.0005	0.0047
neg_251 1	Tyrosyl-Proline	down	277.1192	2.89185	C14H18 N2O4	-	61.6	-0.506 9	-	1.0406	1.0666	0.9565	0.0129	0.0455
neg_262 2	Leukotriene D4	up	531.2302	3.197883333	C25H40 N2O6S	-	59.4	0.1584	Arachidonic acid metabolism; Metabolic pathways; Neuroactive ligand-receptor interaction; Fc epsilon RI signaling pathway; Asthma	2.0954	2.0569	1.2019	0.0063	0.0272
neg_297 8	AFN911	down	532.2403	4.600416667	C29H33 N7O2	-	79.5	-7.646 1	-	1.1152	1.0991	0.9605	0.0012	0.0082
neg_311	Armillarinin	down	463.1541	5.246083333	C24H29C	-	54.7	2.5184	-	3.2196	3.2375	0.6635	0.0003	0.0037

7					lO7									
neg_3149	1,2-Dehydrosalsolinol	down	353.1502	5.378516667	C10H11NO2	-	65.3	-1.3797	-	2.2258	2.3416	0.7393	0.0030	0.0158
neg_3250	3,4,5-trihydroxy-6-(2-hydroxy-1,2-diphenylethoxy)oxane-2-carboxylic acid	down	389.1241	5.8601	C20H22O8	-	75	-0.2587	-	1.3259	1.3367	0.9445	0.0005	0.0047
neg_3314	Arginyl-Hydroxyproline	down	619.3135	6.0533	C11H21N5O4	-	73.5	-5.9242	-	1.9909	1.9296	0.8612	0.0057	0.0252
neg_3357	6-[(6-{[3-(3,4-dimethoxyphenyl)-7-methoxy-8-methyl-4-oxo-4H-chromen-5-yl]oxy}-3,4-dihydroxy-5-[(3,4,5-trihydroxy-6-methyloxan-2-yl)oxy]oxan-2-yl)methoxy]-3,4,5-trihydroxyoxane-2-carboxylic acid	up	825.2405	6.1158	C37H46O21	-	80.5	-6.5341	-	2.5353	2.5378	1.3278	0.0005	0.0047
neg_3442	11-Hydroxy-9-tridecenoic acid	up	273.1706	6.253016667	C13H24O3	-	54.8	-0.6772	-	1.0326	1.0037	1.0314	0.0003	0.0040
neg_3567	2,2'-(3-methylcyclohexane-1,1-diyl)diacetic acid	up	213.1127	6.39055	C11H18O4	71.4	-	-2.4266	-	1.8156	1.8009	1.1158	0.0000	0.0008

neg_3585	4,5-Dihydrovomifoliol	up	225.1492	6.411116667	C13H22O3	-	50.7	-2.0249	-	2.0708	2.0287	1.1380	0.0000	0.0002
neg_3613	LysoPC(22:6(4Z,7Z,10Z,13Z,16Z,19Z))	down	612.3304	6.4522	C30H50NO7P	-	95.4	-0.4278	Glycerophospholipid metabolism; Choline metabolism in cancer	1.9696	1.9672	0.8488	0.0047	0.0219
neg_3667	2-Arachidonylglycerol	down	801.5511	6.5272	C23H38O4	-	57	-1.4862	Neuroactive ligand-receptor interaction; Retrograde endocannabinoid signaling; Thermogenesis	1.5469	1.5001	0.9319	0.0005	0.0047
neg_3917	PE(20:3/0:0)	down	502.2935	6.821833333	C25H46NO7P	78	-	-0.7888	-	1.2319	1.2325	0.9445	0.0184	0.0582
neg_3966	LysoPC(20:3(5Z,8Z,11Z))	down	590.3456	6.890116667	C28H52NO7P	-	71.2	-1.4411	-	1.3256	1.2964	0.9409	0.0203	0.0623
neg_4017	Hydroxybuprenorphine	up	504.2704	6.972016667	C29H41NO5	-	57.2	-5.6677	-	1.5448	1.5294	1.0866	0.0008	0.0060
neg_5272	Carindone	up	533.2914	8.645016667	C31H44O6	-	86.1	5.7153	-	1.3108	1.2627	1.0438	0.0000	0.0007
neg_5386	2-Palmitoylglycerophosphocholine	up	533.2912	7.85995	C24H51NO7P+	-	76.4	4.5946	-	1.4093	1.3557	1.0603	0.0002	0.0031
neg_5437	1-(11Z-eicosenoyl)-glycero-3-phosphate	down	509.2879	7.632183333	C23H45O7P	-	59.7	-1.2719	-	1.4275	1.4220	0.9528	0.0000	0.0005

neg_5514	1-(11Z,14Z-eicosadienoyl)-glycero-3-phosphate	down	507.2724	7.39005	C23H43O7P	-	53.1	-0.9959	-	1.5610	1.5583	0.9311	0.0007	0.0054
neg_5534	1-eicosanoyl-glycero-3-phosphate	down	511.3033	7.3414	C23H47O7P	-	74.9	-1.7339	-	1.1067	1.0832	0.9475	0.0102	0.0382
neg_5706	LysoPC(18:1(9Z))	down	566.3460	7.005933333	C26H52NO7P	-	65.3	-0.6183	-	1.0654	1.0902	0.9620	0.0365	0.0952
neg_5805	PE(18:1(9Z)/0:0)	down	478.2933	6.890116667	C23H46NO7P	96.9	-	-1.2921	-	1.3639	1.3668	0.9552	0.0003	0.0037
neg_5847	21beta-Hydroxyhederagenin	down	469.3318	6.83555	C30H48O5	-	68.6	-1.1022	-	2.1950	2.1644	0.8498	0.0001	0.0012
neg_5871	2-Hydroxymyristic Acid	up	243.1962	6.8081	C14H28O3	69.6	-	-1.5368	-	1.1259	1.1125	1.0552	0.0092	0.0355
neg_5884	LysoPC(20:4(5Z,8Z,11Z,14Z))	down	588.3303	6.787633333	C28H50NO7P	-	73.6	-0.6645	Glycerophospholipid metabolism; Choline metabolism in cancer	1.3441	1.3683	0.9389	0.0188	0.0592
neg_5897	(Z)-6-Nonenal	down	279.2326	6.780766667	C9H16O	-	72.6	-1.0974	-	1.1497	1.1432	0.9542	0.0048	0.0222
neg_5936	PE(22:6/0:0)	down	524.2779	6.725883333	C27H44NO7P	52.1	-	-0.6126	-	1.6995	1.6718	0.9056	0.0082	0.0325
neg_5986	DG(8:0/16:0/0:0)	up	477.3582	6.684733333	C27H52O5	-	78.3	4.5211	-	1.3031	1.2867	1.0794	0.0095	0.0362

neg_6035	(3beta,5alpha,6alpha,9alpha,22E,24R)-Ergosta-7,22-diene-3,5,6,9-tetrol	down	491.3373	6.643216667	C28H46O4	-	72.5	-1.1530	-	1.0647	1.0481	0.9611	0.0071	0.0293
neg_6206	Traumatic Acid	up	227.1284	6.4522	C12H20O4	75.3	-	-2.0241	alpha-Linolenic acid metabolism; Biosynthesis of secondary metabolites	1.5154	1.5011	1.0770	0.0002	0.0024
neg_6215	(3beta,17alpha,23R)-17,23-Epoxy-3,29-dihydroxy-27-norlanost-8-ene-15,24-dione	down	517.3168	6.438483333	C29H44O5	-	83.8	-0.5162	-	1.1256	1.0695	0.9700	0.0009	0.0070
neg_6218	2,3-dinor Prostaglandin E1	up	325.2017	6.438483333	C18H30O5	71.6	-	-1.0893	-	1.0298	0.9930	1.0332	0.0010	0.0072
neg_6285	6-[(2-{[3-(3,4-dimethoxyphenyl)-7-methoxy-8-methyl-4-oxo-4H-chromen-5-yl]oxy}-5-hydroxy-6-(hydroxymethyl)-3-[(3,4,5-trihydroxy-6-methyloxan-2-yl)oxy]oxan-4-yl)oxy]-3,4,5-trihydroxyoxane-2-carboxy	up	825.2404	6.363133333	C37H46O21	-	63.8	-6.6038	-	2.3094	2.3413	1.2696	0.0032	0.0167

lic acid

neg_6291	1b-Hydroxycholic acid	down	847.5578	6.356266667	C24H40O6	-	61	0.1340	-	2.0416	2.0427	0.9061	0.0000	0.0000
neg_6296	2-Hydroxy-2,6,6-trimethylcyclohexanone	up	201.1126	6.356266667	C9H16O2	-	71.7	-3.7776	-	1.3996	1.3618	1.0561	0.0001	0.0016
neg_6366	11-Hydroxyeicosatetraenoate glyceryl ester	down	439.2698	6.29455	C23H38O5	-	58.8	-0.8095	-	1.1579	1.1631	0.9719	0.0000	0.0001
neg_6570	Monomenthyl succinate	up	301.1655	6.032666667	C14H24O4	-	53	-0.7700	-	1.8237	1.8482	1.1054	0.0000	0.0005
neg_6605	(1beta,2beta,5beta)-p-Menth-3-ene-1,2,5-triol	up	231.1234	5.956316667	C10H18O3	-	69.1	-2.2559	-	1.1633	1.1198	1.0410	0.0001	0.0018
neg_6653	2,3-dimethoxy-4-[(2E)-3-phenylprop-2-en-1-yl]phenol	up	315.1237	5.729183333	C17H18O3	-	53.1	-0.2515	-	1.1846	1.1905	1.0397	0.0001	0.0020

neg_6727	Auxin b	down	347.1610	5.4245	C18H30O4	-	73.9	-6.6232	-	1.8414	1.7631	0.8633	0.0176	0.0564
neg_6744	N-(4-aminobutyl)-3-[3-methoxy-4-(sulfooxy)phenyl]prop-2-enimidic acid	up	325.0861	5.359933333	C14H20N2O6S	-	55.4	-0.8861	-	2.4100	2.4191	1.3586	0.0031	0.0162
neg_6782	Suberic acid	up	173.0812	5.212966667	C8H14O4	72.2	-	-4.1482	-	1.5174	1.5156	1.0638	0.0000	0.0003
neg_6787	3,4,5-trihydroxy-6-{3-hydroxy-5-[2-(2-hydroxyphenyl)ethyl]phenoxy}oxane-2-carboxylic acid	down	405.1191	5.185716667	C20H22O9	-	74.2	0.0534	-	1.5660	1.5396	0.9263	0.0004	0.0047
neg_6789	Tyrosyl-Tryptophan	up	348.1352	5.176716667	C20H21N3O4	-	78.6	-0.5113	-	1.7824	1.7753	1.1254	0.0009	0.0065
neg_6899	2-(hydroxymethyl)-2-methyl-2H-chromen-5-ol	up	237.0763	4.7508	C11H12O3	-	75.4	-2.6836	-	2.0778	2.0079	1.1329	0.0000	0.0008
neg_6945	6-methoxy-3-(4-methoxyphenyl)-3,4-dihydro-2H-1-benzopyran-7-ol	up	331.1185	4.537583333	C17H18O4	-	68.9	-0.6470	-	1.1083	1.1036	1.0351	0.0002	0.0023
neg_6969	L-Hexahydro-3-imino-1,2,4-oxadiazepine-3-carboxylic acid	up	317.1240	4.403783333	C5H9N3O3	-	73.6	7.7353	-	1.1122	1.1483	1.0505	0.0180	0.0574

neg_7072	Gynocardin	up	284.0774	3.969966667	C12H17NO8	-	59.5	-0.7019	-	2.4626	2.4236	1.4410	0.0226	0.0673
neg_7277	Nonate	up	233.1026	3.31685	C9H16O4	-	70.8	-2.2658	-	1.1143	1.1268	1.0422	0.0005	0.0047
neg_7564	Ethyl glucuronide	up	203.0556	2.7671	C8H14O7	-	74.8	-2.3084	-	1.8948	1.8734	1.1347	0.0000	0.0009
neg_7792	Phenylacetylglycine	up	238.0716	2.2415	C10H11NO3	-	50.2	-2.3377	Phenylalanine metabolism	1.4361	1.4237	1.0639	0.0000	0.0004
neg_8194	Ascorbigen	up	326.0645	0.827216667	C15H15NO6	-	54	-0.4192	-	3.6600	3.5896	1.6443	0.0000	0.0000
neg_8312	3-Oxoadipic acid	up	205.0345	0.799733333	C6H8O5	-	56.3	-5.7016	Metabolic pathways; Benzoate degradation; Microbial metabolism in diverse environments; Degradation of aromatic compounds	1.3423	1.3289	1.0735	0.0024	0.0135
neg_8459	2-Hydroxyethanesulfonate	up	124.9905	0.737966667	C2H6O4S	-	52.8	-7.4877	Metabolic pathways; Taurine and hypotaurine metabolism	1.6999	1.6593	1.1012	0.0000	0.0002
neg_8507	Galactose 1-phosphate	down	241.0114	0.703883333	C6H13O9P	-	62.6	-1.7778	Metabolic pathways; Amino sugar and nucleotide sugar metabolism; Galactose metabolism	1.6497	1.5483	0.8993	0.0278	0.0784
neg_897	Lacto-N-biose I	up	418.1119	0.551533333	C14H25	-	75.3	-0.779	-	1.0852	1.0574	1.0480	0.0063	0.0273

9 NO11 4

neg_8333	Isocitrate	up	191.0191	0.792866667	C6H8O7	54.2	-	-3.4614	Metabolic pathways; Glyoxylate and dicarboxylate metabolism; Biosynthesis of secondary metabolites; Central carbon metabolism in cancer; Microbial metabolism in diverse environments; 2-Oxocarboxylic acid metabolism; Biosynthesis of plant hormones; Carbon fixation pathways in prokaryotes; Biosynthesis of amino acids; Carbon metabolism; Glucagon signaling pathway; Citrate cycle (TCA cycle); Biosynthesis of alkaloids derived from terpenoid and polyketide; Biosynthesis of alkaloids derived from histidine and purine; Biosynthesis of alkaloids derived from ornithine, lysine and	1.5979	1.5843	1.0735	0.0000	0.0003

nicotinic acid;
Biosynthesis of alkaloids derived from shikimate pathway; Biosynthesis of terpenoids and steroids; Biosynthesis of phenylpropanoids; Biosynthesis of plant secondary metabolites

neg_414	Malic acid	up	133.0134	0.8621	C4H6O5	78.2	-	-7.1445	Biosynthesis of secondary metabolites; Renal cell carcinoma; Carbon metabolism; Pyruvate metabolism; Citrate cycle (TCA cycle); Glyoxylate and dicarboxylate metabolism; Proximal tubule bicarbonate reclamation; Carbon fixation in photosynthetic organisms; Glucagon signaling pathway; Biosynthesis of alkaloids derived from terpenoid and polyketide; Biosynthesis of alkaloids derived from histidine and purine; Taste transduction; Biosynthesis of alkaloids derived from shikimate pathway; Biosynthesis of terpenoids and steroids; Biosynthesis of phenylpropanoids; Biosynthesis of plant	1.3036	1.2972	1.0366	0.0000	0.0000

secondary metabolites; Metabolic pathways; Microbial metabolism in diverse environments; Pathways in cancer; Methane metabolism; Biosynthesis of alkaloids derived from ornithine, lysine and nicotinic acid; Carbon fixation pathways in prokaryotes; Two-component system; Central carbon metabolism in cancer; Biosynthesis of plant hormones

neg_2911	N'-Formylkynurenine	up	271.0471	4.273883333	C11H12N2O4	-	61.4	-8.2997	Metabolic pathways; Tryptophan metabolism	1.1671	1.1306	1.0515	0.0083	0.0330
